# Chlorpyrifos Disrupts Acetylcholine Metabolism Across Model Blood-Brain Barrier

**DOI:** 10.3389/fbioe.2021.622175

**Published:** 2021-08-27

**Authors:** Dusty R. Miller, Ethan S. McClain, James N. Dodds, Andrzej Balinski, Jody C. May, John A. McLean, David E. Cliffel

**Affiliations:** ^1^ Department of Chemistry, Vanderbilt University, Nashville, TN, United States; ^2^ Center for Innovative Technology, Vanderbilt University, Nashville, TN, United States; ^3^ Vanderbilt Institute of Chemical Biology, Vanderbilt University, Nashville, TN, United States; ^4^ Vanderbilt-Ingram Cancer Center, Vanderbilt University, Nashville, TN, United States; ^5^ Vanderbilt Institute for Integrative Biosystems Research and Education, Vanderbilt University, Nashville, TN, United States

**Keywords:** organophosphate, mass spectrometry, organ-on-a-chip, electrochemistry, pesticide

## Abstract

Despite the significant progress in both scientific understanding and regulations, the safety of agricultural pesticides continues to be called into question. The need for complementary analytics to identify dysregulation events associated with chemical exposure and leverage this information to predict biological responses remains. Here, we present a platform that combines a model organ-on-chip neurovascular unit (NVU) with targeted mass spectrometry (MS) and electrochemical analysis to assess the impact of organophosphate (OP) exposure on blood-brain barrier (BBB) function. Using the NVU to simulate exposure, an escalating dose of the organophosphate chlorpyrifos (CPF) was administered. With up to 10 μM, neither CPF nor its metabolites were detected across the BBB (limit of quantitation 0.1 µM). At 30 µM CPF and above, targeted MS detected the main urinary metabolite, trichloropyridinol (TCP), across the BBB (0.025 µM) and no other metabolites. In the vascular chamber where CPF was directly applied, two primary metabolites of CPF, TCP and diethylthiophosphate (DETP), were both detected (0.1–5.7 µM). In a second experiment, a constant dose of 10 µM CPF was administered to the NVU, and though neither CPF nor its metabolites were detected across the BBB after 24 h, electrochemical analysis detected increases in acetylcholine levels on both sides of the BBB (up to 24.8 ± 3.4 µM) and these levels remained high over the course of treatment. In the vascular chamber where CPF was directly applied, only TCP was detected (ranging from 0.06 μM at 2 h to 0.19 μM at 24 h). These results provide chemical evidence of the substantial disruption induced by this widely used commercial pesticide. This work reinforces previously observed OP metabolism and mechanisms of impact, validates the use of the NVU for OP toxicology testing, and provides a model platform for analyzing these organotypic systems.

## Introduction

Organophosphates (OPs) are a class of compounds commonly used in commercial pesticides (e.g., parathion, chlorpyrifos, and diazinon) but also include nerve gas chemical warfare agents such as sarin, VX, and Novichok agents. While OPs are widely used throughout the world for insect control, concerns about their toxicity to humans and animals led to restrictions in the United States for residential use in 2001. In 2018, a United States federal appeals court ordered the United States Environmental Protection Agency to completely ban the use of the broad-spectrum organophosphate pesticide chlorpyrifos (CPF) based in part on epidemiological studies linking prenatal CPF exposure to neurobehavioral deficits in children (Rauh et al., 2011; Rauh et al., 2012). To gain additional insights into CPF-induced chemical and morphological perturbations, *in vitro* organotypic models offer medium-throughput systems that complement traditional cell culture techniques and may replace or reduce animal testing (Pridgeon et al., 2018; Low et al., 2021). These organotypic models aim to replicate human physiology and provide the experimental flexibility necessary to address the effects of OPs on human health (Nolan et al., 1984; Marín-Padilla, 2012; Shamir and Ewald, 2014; Bruner-Tran et al., 2017; Vernetti et al., 2017; Theobald et al., 2018).

**FIGURE 1:**
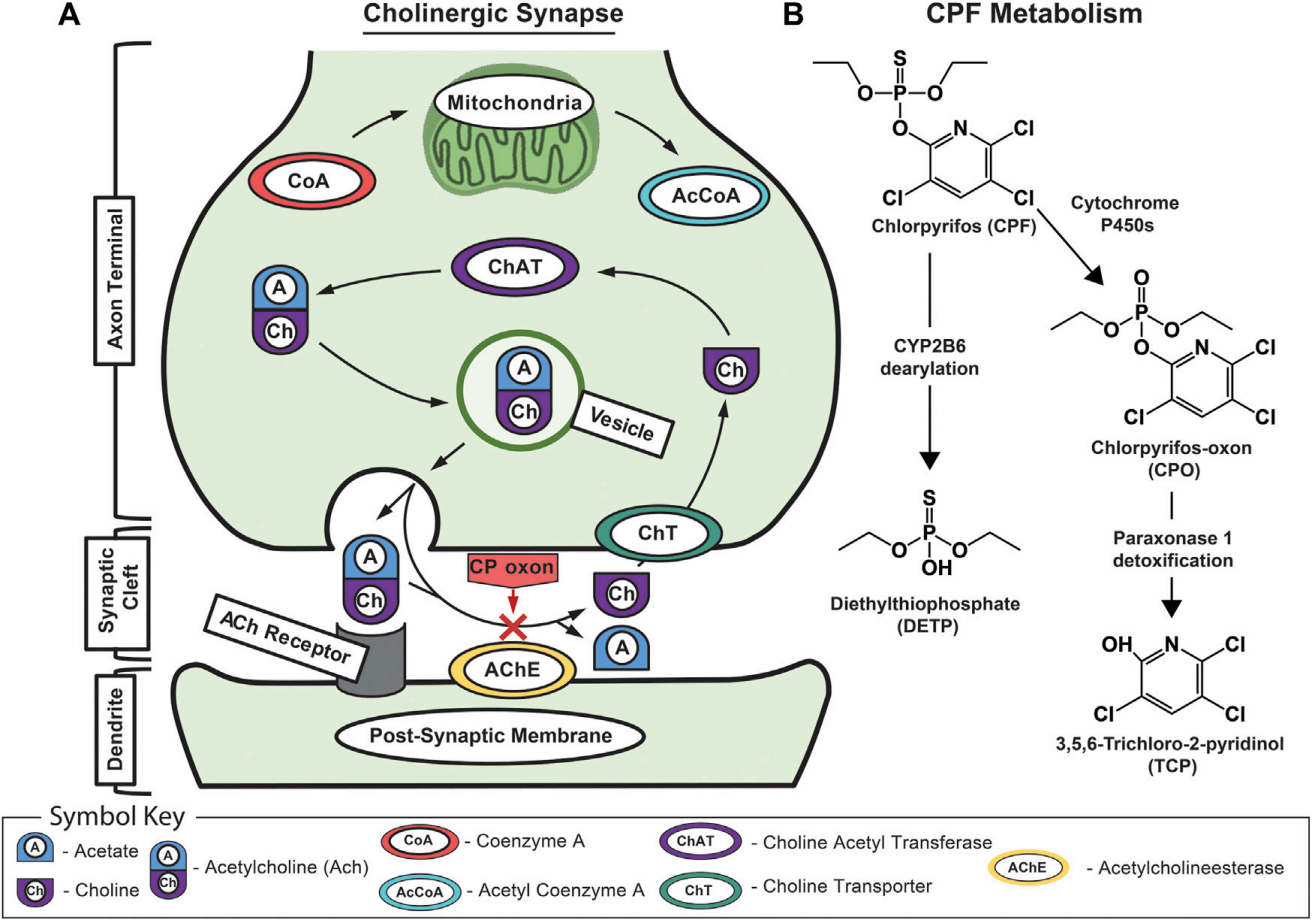
Schematic of acetylcholine and chlorpyrifos biochemistry. **(A)** Schematic of the mechanism of CPF toxicology at the cholinergic synapse showing normal signaling where acetylcholine is recognized by the acetylcholine receptor on the dendrite’s postsynaptic membrane before being rapidly broken down by acetylcholinesterase into acetic acid and choline. Normally, the free choline is taken back up into the presynaptic neuron where choline acetyltransferase turns it back into acetylcholine before it is packaged into vesicles for subsequent release. The red “X” indicates CPO-induced inhibition of acetylcholinesterase that leads to a buildup of acetylcholine in the synaptic cleft and, eventually, excitotoxicity, neuropathy, and death. **(B)** The major metabolic pathway for CPF bioactivation, dearylation, and biodegradation showing associated primary metabolites including CPF; chlorpyrifos oxon (CPO); diethylthiophosphate (DETP); and 3,5,6-trichloro-2-pyridinol (TCP).

The primary mechanism of CPF neurotoxicity is through the inhibition of acetylcholinesterase, yet its full metabolic response remains unclear. At a cellular level, cholinergic signal transmission is accomplished by acetylcholine release into the neuronal synapse before it is broken down by acetylcholinesterase and taken back up by presynaptic neurons (Figure 1; Taylor et al., 1999). OPs inhibit acetylcholinesterase by binding to serine in the active site, preventing acetylcholine from interacting with the enzyme (Mileson et al., 1998; Prendergast et al., 1998; Karanth et al., 2006). Before binding, CPF is metabolically converted by cytochrome P450s into the bioactive chlorpyrifos oxon (CPO) form. When acetylcholinesterase is inhibited, acetylcholine can accumulate in motor neuron synapses causing excitotoxicity, seizures, and brain damage (Prendergast et al., 1998; Yang et al., 2001; Timchalk et al., 2005; Slotkin, 2011). OP neurotoxicity can also extend to necrosis, apoptosis, and oxidative stress-mediated pathways (Carlson et al., 2000; Kashyap et al., 2010; Moore et al., 2010; Kashyap et al., 2011; Kashyap et al., 2013; Park et al., 2015a). Mice and rats are considered standard models for controlled toxicological studies although historical studies include human volunteers (Levin et al., 2001). In mice, CPF has been shown to cause alterations to the integrity of the BBB upon exposure, enabling CPF and other toxicants to enter the brain (Levin et al., 2001; Li and Ehrich, 2013). These risks associated with CPF exposure combined with its continued use in the United States demand further investigation and refinement of our ability to identify dysregulation events associated with chemical exposure and leverage this information to predict biological responses (Rauh et al., 2012; Smith et al., 2014).

In developing models for studying BBB toxicity, organs-on-chips offer several advantages (Cucullo et al., 2011; Griep et al., 2013; Prabhakarpandian et al., 2013; Adriani et al., 2017; Phan et al., 2017; Wang et al., 2017; Maoz et al., 2018). Built upon the knowledge gained from early experiments with cocultures and Transwells, the development of organ-on-chip technologies aims to combine the versatility of *in vitro* experimentation with cutting-edge engineering and analytics to refine the questions that can be addressed (Parran et al., 2005; Balbuena et al., 2010; Daneman and Prat, 2015; Hopkins et al., 2015; Helms et al., 2016; Voorhees et al., 2017; Zhang et al., 2017; Grebenyuk and Ranga, 2019). Organs-on-chips vary in construction but all contain three-dimensional supports that spatially orient cultures to develop organ-like qualities (Dingle et al., 2015; Adriani et al., 2017; Soscia et al., 2017). Recent advances include modifying the physical dimensions and mechanical properties by incorporating gels or matrices, encouraging the production of an extracellular matrix (ECM), and investigating novel materials (Tang-Schomer et al., 2014; Jeong et al., 2015; Lozano et al., 2015; Hutson et al., 2016; Zhuang et al., 2018; Zhuang et al., 2018; Grebenyuk and Ranga, 2019). Within these structures, perfusion of media enables the exchange of nutrients and metabolites and provides the shear stress needed to stimulate cell proliferation and differentiation. Perfusion has been driven by gravity, pneumatic, piezoelectric, or mechanical systems (Takayama et al., 1999; Araci and Quake, 2012; Brown et al., 2015; Park et al., 2015b; Fernandes et al., 2016; Koo et al., 2018; Wang et al., 2018; Balaji et al., 2018). The miniaturization of these features reduces the quantity of reagents used, thereby decreasing cost and supporting the incorporation of cells that are either difficult to culture or difficult to isolate (Herculano-Houzel, 2009; Volpatti and Yetisen, 2014; Mohammed et al., 2015; DiMasi et al., 2016; Bang et al., 2017; Campisi et al., 2018). Additionally, efforts to instrument these chips can provide real-time, nondestructive measurements of these systems (Booth and Kim, 2012; Griep et al., 2013; Kilic et al., 2016). For example, neuron excitability can be studied by integrating organ-on-chip technology with electrodes to both stimulate and report the burst-firing frequency rate and power (Hasan and Berdichevsky, 2016; Soscia et al., 2017). Perhaps the most important role for organotypic cultures resides in their application in toxicology, supplementing preclinical cell culture methods, and reducing animal testing (Nolan et al., 1984; Low et al., 2021). There are now a wide range of platforms available with a high degree of specialization allowing researchers to ask detailed questions about BBB health and disease (Lancaster et al., 2013; Banerjee et al., 2016; Kilic et al., 2016; Adriani et al., 2017).

Recently, Wikswo and colleagues developed a neurovascular unit (NVU), an organotypic model that approximates the human BBB, by creating a paracellular barrier comprised of endothelial cells, astrocytes, and pericytes and seeding it with neurons (Brown et al., 2015). This NVU has been shown to be a useful model to assess both acute (seconds to minutes) and chronic (days to weeks) toxic exposure (Brown et al., 2015; Brown et al., 2016). The dual-chamber NVU design—a neuronal (2.9 µL) and a vascular (17.5 µL) section—is equipped with independent microfluidic perfusion control so that environmental exposure can be simulated by administration of toxicants to the vascular side while analyzing the neuronal side for metabolic changes and for the infiltration of toxicants that breach the engineered BBB. Furthermore, these two chambers can be seeded with as little as twenty thousand cells, making the NVU feasible for culturing rare or difficult to isolate cells. Taken together, these features make the NVU well suited for transbarrier analysis of OP exposure and, with careful consideration, as a regulatory tool for toxicology (Fennema et al., 2013; Andersen, 2014; Schadt et al., 2014; Koo et al., 2018; Wang et al., 2018; Pimentel et al., 2020; Raimondi et al., 2020).

This work presents a platform for simulating and analyzing toxicological events that supports the prediction of biological responses through morphological and metabolic analysis. NVUs seeded with the four cell types necessary for proper BBB function were cultured and the vascular side of these NVUs was then dosed with the organophosphate CPF, simulating environmental exposure. Eluate from the vascular and neuronal sides was assessed using liquid chromatography coupled to tandem mass spectrometry (LC-MS/MS) for targeted toxicant profiling and electrochemical analysis for targeted metabolite profiling. These data validate the predictive power of the NVU, the high analytical utility of combined MS, and electrochemical measurements and provide insight into the substantial disruption induced by this widely used commercial pesticide. Applying this unique platform with expanded analytics is an important advance in studying OP toxicity.

## Materials and Methods

***NVU Bioreactor Fabrication****.* The NVU bioreactor was designed for independent perfusion of the two chambers and is described in detail elsewhere (Brown et al., 2015), with some minor modifications. The NVU is a two-chamber device made with three layers of polydimethylsiloxane (PDMS) separated by a 0.4 µM pore polyethylene terephthalate (PET) membrane (Fisher Scientific, Hampton, NH). First, the neuronal layer and the vascular layer were created by pouring 2.5 and 16g, respectively, of PDMS precursors (10:1 wt:wt ratio of base:curing agent, Sylgard 184, Dow Corning) into encapsulated wafers, cured (65°C, 4 h), and demolded. To fabricate the middle layer, 30 g of PDMS precursors was poured into the middle layer mold with spacers and placed in a dish. The dish was covered and placed under vacuum until bubbles formed and repressurized and the process was repeated. The dish was then removed from vacuum and bubbles were blown off. To control for layer thickness, the top mold of the middle layer was then placed on the spacers with weights (≤70g) on top and allowed to cure at room temperature for 48 h. After drying, excess PDMS was trimmed off and the middle layer was removed and cured at 65°C for 2 h.

With all three layers cured and trimmed to size, the NVU could be assembled. First, the vascular layer and a glass plate (50 mm × 75 mm) were both plasma-activated (40 s, high power setting, air metered into vacuum, Harrick Plasma Cleaner, Ithaca, NY) and brought together to bond with the chamber facing upwards. Meanwhile, the neuronal layer was punched with inlet and outlet ports (Miltex 1.5 mm OD, Integra York, Inc., York PA) to accommodate microfluidic perfusion. Both the middle and neuronal layers were plasma-activated and bonded together with the neuronal layer channels facing the middle layer, and the assembly was placed in a 65°C oven for 10 min to complete the bonding process. After bonding, both the middle-neuronal and the vascular-plate layer assemblies were annealed at 200°C for 4 h. PET membranes were plasma-activated and immersed in an 80°C solution of 0.2% bis-amino (Sigma Aldrich, St. Louis, MO) and 0.1% deionized water in IPA. After drying, these membranes were placed in 70% ethanol (30 min, room temperature) and blown dry with filtered N_2_. The membranes were then brought together with the vascular layer to bond, and the neuronal-middle layer was added by facing the middle layer to the membrane so that the reliefs in the PDMS-created chambers overlapped completely with one another except for the inlet and outlet ports. The assembled device was placed in the oven (60°C) overnight to finish curing. Completed NVU vascular and neuronal chambers (2.9 and 17.5 µL, respectively) each incorporated an entrance and exit port to facilitate independent perfusion. A noteworthy change from previous versions of this device fabrication is the incorporation of 0.4 µM pore PET membrane that is more transparent and enhances imaging (previous iterations of this device used a polycarbonate membrane). The transition to PET membranes was done because of the manufacturer’s change in membrane properties that made them unsuitable for the NVU and also to increase visibility for enhanced microscopy. All NVU devices were packaged and gamma sterilized overnight (Mark 1 Cesium Irradiator, Glendale, CA) before use. Schematics of the NVU were made to scale in CAD with the help of the Vanderbilt Institute for Integrative Biosystems Research and Education.

***PDMS Absorption of CPF****.* To determine the extent to which CPF is absorbed into PDMS, a floating disk experiment was conducted as before with some modifications (Auner et al., 2019). First, 3 mm thick medical grade PDMS (same as that used in the NVU fabrication) was cut with a biopsy punch (diameter = 6 mm) to form disks. Using a 20 mM stock solution of CPF made in DMSO, 25, 50 75, and 100 µM CPF solutions were made in 10 mM SDS (to aid solubility). Then, each disk (3) was submerged in 2 ml of 100 µM CPF solution inside of a 4 ml glass vial and the absorption of this solution as well as that of calibrants (25, 50, 75, and 100 µM CPF) was measured every hour at 290 nm. From these data, the concentration of CPF still in solution was calculated.

***Harvesting Primary Rat Neurons****.* Primary rat neurons were harvested as previously described with some modifications (McKenzie et al., 2012). Briefly, pure neuronal cultures were obtained from embryonic day 18 Sprague-Dawley rats. To obtain primary rat neurons, brains from anesthetized rats were dissected, and cortices were trypsinized (Sigma, United States) before being transferred to 10 ml of neurobasal media (Sigma). This solution was then strained (40 µm), counted, and centrifuged (1,000 RCF, 5 min). The resulting pellet was resuspended [neurobasal media, 10% DMSO (Sigma), at either 7.5 × 10^5^ or 5 × 10^8^ cells/ml] and frozen down until needed for either plating in well plates or NVU seeding, respectively.

***Staining Primary Rat Neurons****.* Neurons were stained to confirm the presence of cholinergic neurons in culture. Neurons stored at −80°C at 750,000 cells/ml were plated 2 ml/6 well Transwell plates coated with polyornithine and maintained in plating media [Dulbecco’s modified Eagle’s medium (DMEM) media (Gibco) with 8% F12, 8% fetal bovine serum (FBS), 80 μM L-glutamate, and 1% penicillin/streptomycin (Fisher)] for days *in vitro* (DIV) 0–13. During this time, the cells were maintained with half media changes every 2–3 days. Two weeks after harvest, the neurons had a half media change to D2C media (94% DMEM, L-glutamine, 2% FBS, 0.025 M HEPES, 0.0125 mM L-glutamine, 24 U/mL penicillin, and 24 μg/ml streptomycin), and two drops of AraC were added through a plugged pasture pipette for a final concentration of 1–2 M/well. From then on, cells were maintained with D2C media until use. Neurochemical staining for choline acetyltransferase (ChAT), Neuron Specific Tubulin (NST), and 4′,6-diamidino-2-phenylindole (DAPi) to stain all cells merged to show overlap as done before with some modifications (Lizama et al., 2018).

***Neuronal Response to CPF****.* Neurons were either treated with a media change control or 100 µM CPF for 18 h and imaged as before (Lizama et al., 2018). Experiments were conducted at 37°C and 5% CO_2_.

***Cell Culture****.* Endothelial cells, astrocytes, and pericytes were cultured as before with some modifications (Brown et al., 2016). Primary human brain microvascular endothelial cells (hBMVECS, Applied Cell Biology, Kirkland WA, United States) were maintained in endothelial basal media 2 (EBM2, Lonza, Basa, Switzerland) containing 5% FBS, growth bullet kit (hEGF, hydrocortisone, GA-1000 [gentamicin and amphotericin-B], VEGF, hFGF-B, R3-IGF-1, ascorbic acid, and heparin), and 1% penicillin/streptomycin (complete EBM-2). Human astrocytes from brain tissue of a first trimester fetus and SV40 transformed (SVG p12, ATCC, Manassas, VA, United States) and pericytes isolated from human brain tissue (Cat # 1200, ScienCell, Carlsbad, CA, United States) were maintained in a 1:1 DMEM and F-12 with 10% FBS (Allt and Lawrenson, 2001). Endothelial cells, astrocytes, and pericytes were maintained in T-25 flasks (Fisher) under standard culture procedures until collected (passage three) for seeding into the NVU.

***NVU Seeding and Culturing****.* The NVU supported the growth of all four cell types necessary for proper BBB function and is described in detail previously with some modifications (Brown et al., 2015). Prior to cell seeding, NVU devices were first coated with poly-D-lysine (10 μg/ml) in carbonate buffer (0.2 M, pH 9.6, 37°C, 4 h, Fisher) followed by coating with fibronectin and collagen IV (both 400 μg/ml, overnight, 37°C, Sigma). After washing the devices with warm complete EBM-2 media to remove any unbound fibronectin or collagen, endothelial cells were loaded into the vascular chamber (5–10 × 10^6^ cells/ml). The NVU was then placed vascular side up and incubated overnight (37°C, 5% CO_2_). The next day, the vascular chamber was connected to a syringe pump (Harvard Apparatus) and perfused under low flow conditions (2 μL/min) with complete EBM-2 media for 9 days. After this time, the neuronal chamber was loaded with astrocytes (6 × 10^6^ cells/ml) and pericytes (1 × 10^6^ cells/ml) and the device was placed neuronal chamber side up (37°C, 5% CO_2_) to allow the cells to settle and adhere for 2 h before flow was restarted. After culturing these three cell types together for 2 days under low flow conditions, primary rat neurons were loaded into the neuronal chamber (10 × 10^6^ cells/ml) and allowed to attach for 2 h before flow was again restarted. Within each NVU, all four cell types were cultured for 3 days before these devices were ready for leakage and toxicological testing. During the course of these experiments, sister cultures of the cells loaded into the NVU devices were plated and no contamination was detected.

To ensure proper barrier formation, leakage across the engineered BBB was tested using 10 kDa fluorescein isothiocyanate-dextran (FITC-dextran, Sigma) as previously described (Brown et al., 2016). Briefly, FITC-dextran was prepared (100 nM, cell culture media) and administered to the vascular compartment of the NVU (23 h, 2 μL/min), allowing the FITC-dextran to diffuse across the BBB and into the neuronal chamber, reflecting barrier permeability. The effectiveness of the BBB was evaluated by measuring the fluorescent intensity in the neuronal side eluate using a plate reader (TECAN M1000). The permeability, P, was calculated from the FITC concentration using Eq. 1 where V_r_ is the volume in the receiving chamber, t is the time of the experiment, A is the area of the membrane (0.29 cm^2^), C_i_ is the initial concentration, and C_f_ is the measured concentration.

$$\begin{array}{c} P=\frac{V_{r}\times C_{f}}{C_{i}\times A\times t} \end{array}$$ (1)

The permeability of the NVU device with no cells was also measured for comparison. This device was irradiated and soaked in water but otherwise bare. The control device was treated with DMSO as described below in *NVU Treatment With Chlorpyrifos*.

***NVU Treatment With Chlorpyrifos****.* In normal operation, both the vascular and neuronal side chambers of each NVU were perfused with media under low flow (2 μL/min) to maintain the viability of the cell layers that comprise the engineered BBB. To investigate the effects of OPs on the engineered BBB, either CPF (Sigma) or vehicle (dimethyl sulfoxide, DMSO, Sigma) was introduced to the vascular chamber and eluate exiting both vascular and neuronal chambers was collected to be analyzed for biochemical changes. Two experimental setups were executed with this approach: an escalating dose experiment and a time course experiment.

The dose escalation experiment was conducted with varying concentrations of CPF (0, 1, 3, 10, 30, and 100 µM) and six NVU devices (two control devices and four test devices). First, CPF was dissolved in DMSO to 200 mM, filter-sterilized (0.22 µm membrane, Sigma), and stored at −80°C until use. Then, 1 µM CPF was added to complete EBM-2 media and perfused on the vascular side of each test device for 2 h. After collecting that effluent, the syringe containing the 1 µM CPF was exchanged for one containing 3 µM CPF and the process was repeated in this way for all of the CPF concentrations tested. The control NVU was treated with vehicle alone. Eluate samples (240 µL) were collected from both the vascular and neuronal chamber of all NVUs immediately prior to administering each dose and stored at −80°C prior to analysis.

After the escalating dose experiment was conducted, a 10 µM dose of CPF was chosen to investigate the effects of exposure time. A total of five NVU devices (three treated and two control) were prepared and used for the exposure experiments described herein. Additionally, the NVU devices were first perfused with serum-free EBM-2 media before CPF exposure (although the other supplements and growth factors that contribute to the complete media formulation, e.g., hFGF-B, VEGF, R3-IGF, hEGF, GA-1000, ascorbic acid, and hydrocortisone, were added to the media). To add statistical power while reducing the number of NVUs used in the experiment, effluent was collected from the three test devices prior to treatment to serve as a baseline. Two control NVUs were treated with vehicle alone. However, one control device was compromised during the course of the experiment and was subsequently excluded. Therefore, the results between the time-dependent control and the baseline controls were compared for time differences, and after verifying their similarity, the data from both of these controls were pooled for analysis. For the test devices, 10 µM CPF was added to this serum-free media and perfused on the vascular side of each test device. Eluate samples (∼240 µL) from all devices were collected from both the vascular and neuronal chamber of all NVUs at 0, 2, 4, 8, and 24 h and stored at −80°C prior to analysis.

***NVU Microscopy****.* After treatment with CPF or vehicle, NVUs were imaged for morphological analysis. First, live/dead stain was applied to the NVU devices as per manufacturer recommendation (Thermo Fisher) and incubated for 15 min. Fluorescent images of cells stained within the NVU were then collected using an EVOS (Thermo Fisher) automated microscope.

***MS Analysis of NVU Eluate****.* A minimal-handling sample preparation strategy was used, which limited metabolic turnover and degradation while maximizing metabolite recovery. In this strategy, metabolites were extracted from media using a volume of 800 µL of cold (−20°C) methanol added to a 100 µL aliquot of NVU media eluate, vortexed for 30 s, and incubated at −80°C overnight to precipitate proteins. After incubation, samples were cleared by centrifugation (21130 RCF,15 min), and the resulting supernatant was removed and evaporated to dryness in a vacuum concentrator. Dried extracts were reconstituted in 60 μL reconstitution solvent [98:2 (v:v) water:acetonitrile with 0.1% formic acid] for reverse phase LC-MS analysis. The reconstituted samples were then centrifuged (15,000 rpm, 5 min) to remove insoluble debris. Quality control samples were prepared by combining equal volumes (10 μL) of each sample type.

Ultra-high-performance liquid chromatography-mass spectrometry (UHPLC-MS) and multiple reaction monitoring (MRM) were performed on a triple quadrupole mass spectrometer (6,470, Agilent Technologies, Santa Clara, CA, United States) equipped with an Infinity II UHPLC system (1,290, Agilent). Chlorpyrifos and its metabolites were separated on a reverse phase Hypersil Gold RPLC column (1.9 µm, 2.1 mm × 50 mm, Thermo Fisher, Waltham, MA) at ambient temperature. Chromatography was performed at 300 μL/min using solvent A (0.05% formic acid in water) and solvent B (0.05% formic acid in acetonitrile) with the following gradient profile—60% B for 0.5 min, 60–95% B over 3.5 min, and 95–60% B over 0.1 min—and reequilibrated at 60% B over 2 min (gradient length 4.1 min). The injection volume used was 1 μL, with an autosampler temperature of 4°C. The exogenous small molecule, 2,6-di-*tert*-butylpyridine (DtBP), was used as an internal standard. Serial dilutions of DtBP at 10 concentrations were used to determine instrument limits of detection (LOD), quantitation (LOQ), and the calibration curve necessary to convert instrument response to analyte concentrations.

Data acquisition was carried out in fast polarity switching MRM mode using a thermally assisted ESI source (Jet Stream, Agilent) operated with the following conditions: a capillary voltage of 4 kV (positive ion mode) and 2.5 kV (negative ion mode), a nebulizer gas temperature of 300°C with the flow of 8 L/min, and a sheath gas temperature of 300°C with the flow of 11 L/min. Data were acquired using MassHunter Workstation Data Acquisition software (Agilent) and analyzed using MassHunter Quantitative Analysis software (Agilent). A list of metabolites, mass transitions, retention times, and ion polarities used for targeted MS analysis can be found in supplemental materials (Supplementary Table S1). Data represent between four and eight replicate measurements (two–four NVU devices and two technical replicates per sample).

***Acetylcholine Sensor Fabrication and Calibration****.* A screen-printed electrode (SPE) array was enzymatically modified to be selective to acetylcholine (acetylcholine chloride, Sigma) and incorporated into the microclinical analyzer (μCA) microfluidic flow system for automated calibration and analysis as before with some modifications (McKenzie et al., 2015). The μCA consisted of a MicroFormulator and an electrochemical detection cell. The MicroFormulator was designed by and purchased from the Vanderbilt Institute for Integrative Biosystems Research and Education (VIIBRE)/Vanderbilt Microfabrication Core (VMFC) and consisted of a rotary planar peristaltic micropump (US patents 9,874,285 and 9,725,687 and applications claiming priority from US patent application 13/877,925) for delivering flow and a normally closed rotary planar valve (US patent 9,618,129) to select solutions. Microcontrollers and computer software for the MicroFormulator were also purchased from VMFC. The electrochemical detection cell, designed and fabricated by VIIBRE/VMFC, was composed of an electrode array and a microfluidic housing. The electrode array, designed in-house and commissioned for fabrication (Pine Research, Durham, NC), (McKenzie et al., 2015), was composed of five different electrodes: three platinum disk electrodes and two band electrodes. The disk electrodes ($A=1.8\;mm^{2}$) were used for enzymatic detection of acetylcholine. The largest band electrode ($A=19\;mm^{2}$) was silver plated and used as an Ag/AgCl quasireference.

To make the electrodes selective to acetylcholine, a two-enzyme solution of acetylcholinesterase (Sigma) from *Electrophorus electricus* and choline oxidase (Sigma) from *Alcaligenes* was prepared and deposited on the working electrodes. First, each enzyme was dissolved separately to 10 mg/ml in phosphate buffer (2 mM PBS, pH 7, Fisher) containing 800 mg/ml of bovine serum albumin (Sigma) and stored (−18°C) until use. These enzyme solutions were retrieved as required, combined equally by volume, mixed with glutaraldehyde (0.5% wt/v, Sigma), and vortexed (∼5 s). Immediately following vortexing, 1 µL of the mixed enzyme solution was drop-cast onto each working electrode, allowed to air dry for 1 h, and either used immediately or stored (low light, 4°C, 2 mM PBS, 120 mM KCl, pH 7).

The LOD, LOQ, and linearity for the acetylcholine sensor were determined as performed previously (McKenzie et al., 2015; Miller et al., 2018). To incorporate the sensor into the µCA, the SPE was sealed within a polymethylmethacrylate closed-cell housing. The housing was plumbed with Tygon tubing (Cole Parmer, Vernon Hills, IL) to a debubbler (Molecular Devices Inc., Sunnyvale, CA) and attached to a MicroFormulator to facilitate automated calibration and analysis. Calibrations were performed by monitoring the current generated by 21 calibrant solutions (5 μM–5 mM acetylcholine) in buffer (2 mM PBS; 120 mM KCl, pH 7). Calibrants were sampled through a MicroFormulator at a flow rate of 100 μL/min and monitored by a CHI 1,440 potentiostat (CH Instruments, Austin, TX) held at 0.6 V vs. Ag/AgCl with buffer in between to provide a baseline (2 min each). The detection and quantitation limits, along with the sensitivity and linear range of the sensor, were determined by performing a linear regression on the calibration data. The maximum limit of linearity was determined by visual analysis of the calibration curve. The LOD was calculated by dividing three times (10X for LOQ) the error of the blank (y values) by the slope of the determined linear range. Dividing the slope by the area of the disk electrode (1.8 mm^2^) resulted in the sensitivity of the electrode.

***Electrochemical Analysis of Acetylcholine in NVU Eluate****.* The μCA electrochemical detection platform (Miller et al., 2018) was used to determine the acetylcholine levels in NVU samples both with and without CPF treatment. The acetylcholine SPE containing three enzymatically modified acetylcholine sensors was loaded into the µCA housing and the current was monitored by all three to provide technical replicates. Using the MicroFormulator, calibrants were sampled at a flow rate of 100 μL/min (as above, but with six calibrants from 0 to 114 µM). After calibration, NVU eluate was sampled with buffer (2 min, 2 mM PBS, 120 mM KCl, pH 7.0) in between each sample run to establish a baseline. The sensor was recalibrated before and after each NVU sample set to check for sensor degradation/inhibition. The acetylcholine concentration in the sample was determined using the current generated by the sample and the slope and intercept of the calibration curve that was generated by performing a linear regression on the calibration data. *p*-values between sample sets were determined using *t*-test with unequal variance.

## Results and Discussion

This study was designed to assess the utility of the NVU and an electrochemical/MS analytical platform to address three critical aspects of CPF toxicity: 1) How does CPF degrade and what CPF metabolites persist? 2) Does CPF or its metabolites cross the BBB? 3) What effects does CPF exposure have on cellular metabolism at and across the BBB? To this end, environmental exposure to CPF was simulated within the NVU and morphological and metabolic analysis was performed.

To answer these critical questions, it was important to model CPF exposure resulting in significant metabolic disruption without inducing cell death. A few benchmarks for CPF exposure include the United States federally allowed dose of up to 0.03 mg/L of CPF in drinking water (0.085 µM CPF). Detailed murine studies for sublethal doses, report 0.5–5 mg/kg IV doses to result in 10–100 µM CPF in blood (Smith et al., 2012). These dosing levels have been carefully compared to oral administration and historical human studies (Nolan et al., 1984; Smith et al., 2012; Smith et al., 2014). Because CPF binds quickly upon administration, the *in vivo* range of 0–100 µM CPF and a slightly higher dose (300 µM) were chosen for investigation (Lowe et al., 2009).

Using this range of CPF concentrations, we first explored the response of the barrier-forming cells—the endothelial cells—outside of the NVU. Endothelial cells were grown on well plates, exposed to 0, 10, 30, 100, and 300 µM CPF, and visually inspected for morphological changes at 24 h. The treatments resulted in a range of perturbations. The lowest concentration exposure (10 µM) showed slight changes in morphology. At 30μM CPF, treatment was nonlethal but resulted in morphological changes to more circular-shaped cells. The highest exposure tested (300 µM) resulted in the majority of the cells displaying punctate cell morphology and clumping of cells indicating cell death (Supplementary Figure S1). These initial endothelial cell experiments demonstrated varied effects across the range of CPF concentrations. CPF concentrations that were not expected to induce significant cell death in the NVU were tested further (0–100 µM CPF).

Primary neuronal cultures were chosen to ensure that their state of differentiation is representative of mammalian neurons. Specifically, cholinergic neurons were desired to accurately represent susceptibility to OPs. The primary cultures used in this work were 90% neurons and 10% microglia and determined to be 10% positive for choline acetyltransferase, indicating active cholinergic signaling (Supplementary Figure S2). When these neurons were tested for their response to CPF (100 µM), the cells showed a “halo” effect indicating cell death (Supplementary Figure S3). However, if the membrane stays intact, CPF may never reach the neuronal chamber. Historically, research on cholinergic neuronal cultures has been conducted on primary cells, although recently a cholinergic neuronal line was introduced (Ortiz-Virumbrales et al., 2017; Moreno et al., 2018). We are currently working to bring this cholinergic technology into our lab to integrate with the NVU.

The NVU incorporates all four cell types necessary to model BBB function within a three-dimensional, dual-chambered device (Brown et al., 2015; Brown et al., 2016). First, the NVU’s vascular chamber (17.5 µL) was seeded with a human endothelial cell line and grown to confluency. Next, the neuronal chamber (2.9 µL) was seeded with three different cell types: human astrocytic and pericytic cell lines and primary rat neurons (Hamilton, 2010). The neurons were harvested from the forebrain of the rat and the addition of these neurons created a chimeric model fusing a majority of human-derived cells with primary rat neurons. With all four cell types within the device, both chambers were equipped with microfluidic perfusion control in preparation for simulating acute environmental exposure to CPF (device schematic shown in Supplementary Figure S4).

To assess the full-range response of the model BBB to CPF exposure, the NVU was treated with an escalating dose of CPF. A total of five NVU devices were used to serve as biological model replicates. Under perfusion, the vascular sides of each NVU device were exposed to varying concentrations of CPF (0, 1, 3, 10, 30, and 100 µM) in an escalating dose format (successive exposures) over the course of 24 h. Media were collected from both chambers of each NVU prior to each exposure point. Barrier permeability was tested by spiking the vascular side media with 10 kDa fluorescein isothiocyanate-dextran (FITC) and monitoring fluorescence across the barrier (Helms and Brodin, 2014). When an empty device was tested (no cells), the permeability was (2.0 ± 0.4) × 10^−6^ cm/s (Supplementary Figure S5). Compared to the permeability of the empty device (no cells), when the four cell types were added (but before CPF exposure), permeability decreased [0 µM CPF: (0.27 ± 0.05) × 10^−6^ cm/s, control treated with DMSO: (0.28 ± 0.05) x 10^−6^ cm/s]. After exposing the NVUs to 1 µM CPF, the permeability increased [(0.67± 0.27) × 10^−6^ cm/s] and continued to increase upon adding 3 µM CPF [(1.15 ± 0.24) × 10^−6^ cm/s] at which point it stabilized [(1.01 ± 0.46) x 10^−6^ cm/s, 100 µM CPF]. To definitively report the concentration-dependent effects of exposure on permeability without accumulation effects, individual experiments at each concentration are needed. However, this increased permeability indicates that CPF may be able to cross the engineered BBB and enter the neuronal chamber.

To directly investigate the potential crossover of CPF, MS analysis of the media samples was used to track CPF and its primary metabolites at and across the BBB. The canonical pathway for CPF detoxification illustrates that CPF can proceed through the toxic intermediate, CPO, or can be metabolized directly to inactive compounds (Figure 1; Sultatos, 1994). A targeted MS assay, was used to monitor CPF and its metabolites in the escalating dose experiment. MS analysis did not detect CPF nor CPO in either vascular or neuronal eluate samples (Supplementary Figure S6, and numerically in Supplementary Table S2). However, in all conditions in which CPF was administered, TCP—the primary urinary metabolite of CPF—was detected in the vascular effluent, indicating CPF detoxification. At 30 µM CPF and above, TCP was detected across the BBB in the neuronal effluent. At the highest dose of CPF (100 µM), diethylthiophosphate (DETP), another CPF metabolite, was also detected in the vascular effluent. These CPF metabolites are a result of CPF detoxification, which is limited by the enzymatic rate of chlorpyrifos dearylation and/or CPO detoxification (Tang et al., 2006). However, components in the serum used in this experiment such as acetylcholinesterase may have scavenged the CPF and contributed to its lack of detection (Lowe et al., 2009). Still, the observation that neither CPF nor any of the primary metabolites were detected across the BBB with up to 10 µM CPF suggests that barrier integrity was maintained under these conditions. Ultimately, the 10 µM dose—a dose that was thought to have measurable effects in our system without degrading the membrane—was chosen for further investigation.

Before the second NVU experiment was conducted, an electrochemical sensor previously developed by Cliffel and coworkers for the detection of acetylcholine was evaluated for possible interference in this system. The sensor was shown to be sensitive, selective, and stable in the absence of serum (Supplementary Table S4; McClain et al., 2019). Therefore, the second NVU experiment was conducted without serum being added to the media during CPF treatment (although the other components of the Lonza growth bullet kit were added) so that metabolic disruption in specific pathways—such as cholinergic signaling and/or metabolism—could be sensitively and selectively monitored using electrochemical microphysiometry.

**FIGURE 2:**
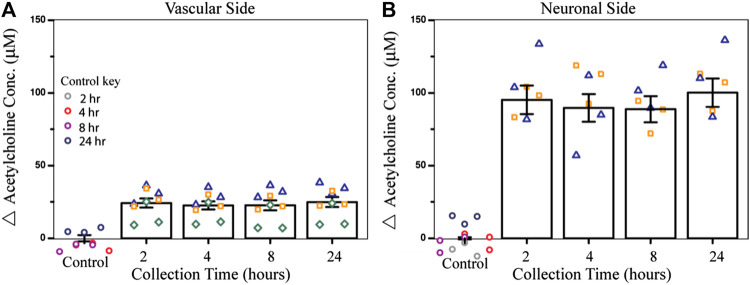
Effect of CPF on acetylcholine metabolism within the NVU as determined from the electrochemical assay. **(A)** Bar graph indicating the change in acetylcholine concentration from control (DMSO) in vascular side eluate over time (2, 4, 8, or 24 h treatment), showing elevated acetylcholine levels (24 ± 3 µM) after 2 h of CPF treatment (10 µM CPF, *p*-value < 0.003). **(B)** Bar graph indicating a change in acetylcholine concentration in neuronal side eluate over time showing elevated acetylcholine (95 ± 10 µM) 2 h after CPF was administered to the vascular side, a significant increase from the control (*p*-value < 0.04). Data are represented as the means and standard errors, symbols represent technical replicates, *n* = 6–9 for samples, and *n* = 9–12 for controls. Control samples were collected over 24 h and their respective collection time is indicated in the control key.

To test the effect of an acute dose of CPF on metabolism within the NVU over time, the vascular sides of the NVUs were dosed with 10 µM CPF and the effluent was electrochemically analyzed. In this study, four NVU devices were used in parallel. When exposed to 10 µM CPF, the vascular eluate exhibited a significant increase in acetylcholine (24 ± 3 μM, *p*-value < 0.003, Figure 2A) after just 2 h and remained elevated for at least 24 h (24 ± 3 µM) compared to control. This acetylcholine buildup is a hallmark of organophosphate poisoning and can interfere with the muscarinic, nicotinic, and central nervous systems causing essential autonomic processes to fail such as respiration and circulation. This acetylcholine buildup may be a result of CPF bioactivation to the ultimate toxicant CPO. Bioactivation is thought to take place primarily in the liver by a cytochrome P450 enzyme (CYP). Within the NVU, a similar CYP produced by the endothelial cells may be responsible for the bioactivation of CPF (Dauchy et al., 2008; Ghosh et al., 2010). Future models of CPF toxicity could include a kidney or liver chip in tandem with the NVU to investigate CYP-dependent effects (Vernetti et al., 2017).

In this same experiment, acetylcholine metabolism was also significantly dysregulated on the neuronal side of the NVU, despite not being treated directly with CPF. Across the BBB, acetylcholine levels increased after 2 h (95 ± 10 μM, *p*-value < 0.04, Figure 2B) and stayed elevated for 24 h (100 ± 10 µM) compared to control. The cholinergic neurons on the neuronal side of the NVU are thought to be primarily responsible for acetylcholine production, perhaps accounting for the even greater change in acetylcholine levels compared to the vascular side. This increase also suggests that either CPF, its toxic metabolites, or other soluble factors were able to cross the BBB and interact directly with the neurons. In future experiments, recently published protocols describing cholinergic neuron differentiation from human-induced pluripotent stem cells (hiPSCs) can be implemented and integrated with this platform so that these results can be compared with those of human cholinergic neurons (McCracken et al., 2014; Paşca et al., 2015; Amin et al., 2016; Moreno et al., 2018; Pas, 2018; Liu et al., 2020).

**FIGURE 3:**
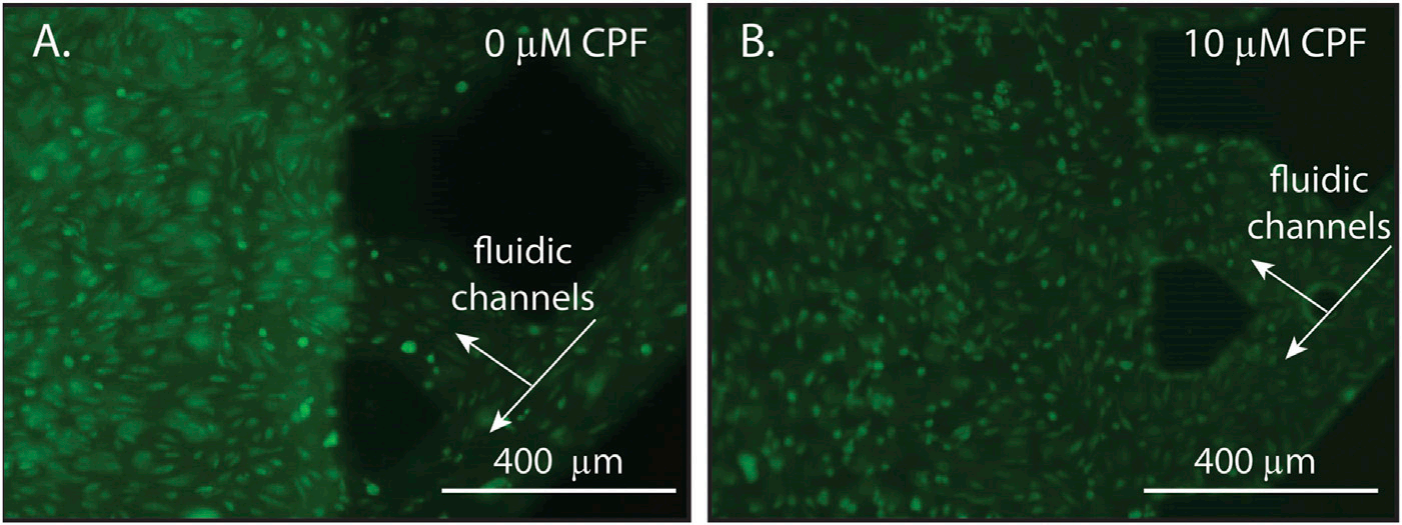
Representative microscopy images of endothelial cells within an NVU following 24 h of continuous exposure with either **(A)** DMSO (vehicle control, *panel A*) or **(B)** CPF (10 μM, *panel B*). The lattice seen at the right of each image is the microfluidic channels of the vascular side of the NVU. **(A)** A control NVU showing evenly dispersed cells with typical morphology, indicating a healthy BBB. **(B)** A CPF-treated NVU showing punctate cell morphology (contracted cells), indicating cellular stress in response to the CPF treatment. For these experiments, the NVUs were perfused with neurobasal media on the neuronal side and EBM2 media on the vascular side either with or without CPF. All cultures were maintained at 37°C and 5% CO_2_.

To investigate how BBB morphology changed over time in response to a long-term CPF exposure at 10 μM, microscopic images were collected after CPF treatment. Treated NVUs displayed some punctate cell morphology (contracted cells) indicative of cellular stress and a compromised BBB, whereas the control NVUs exhibited evenly dispersed cells characteristic of a healthy BBB (Figure 3; Supplementary Figure S7). At moderate levels (1–20 µM), other studies have also found CPF to be tolerated by cells in culture, causing cellular stress but not being directly cytotoxic (chlorpyrifos; Saulsbury et al., 2009; Middlemore-Risher et al., 2011).

**FIGURE 4:**
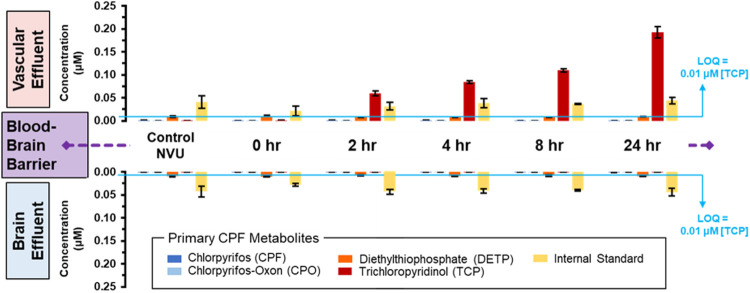
Distribution of CPF and its three primary metabolites at and across the BBB with lengthening exposure times. Using targeted MS, CPF and its metabolites were quantified in both the vascular (**upper plots**) and neuronal (**lower plots**) eluate media samples. These samples were obtained at lengthening durations of exposure to 10 µM CPF within the NVU. The limit of quantitation (LOQ) was determined from serial dilutions of a TCP standard. An internal standard, DtBP, was used to calibrate instrument response during each sample injection.

To directly investigate the potential crossover and metabolism of 10 µM CPF over time, MS was used again to track CPF and its primary metabolites at and across the BBB. Similar to the escalating dose experiment, targeted MS did not detect CPF nor CPO in either vascular or neuronal eluate samples (Figure 4 and displayed numerically in Supplementary Table S2). Only one metabolite, TCP, was detected above the limit of quantitation (∼0.01 µM) and only on the vascular side. TCP was detected at ∼0.05 µM after 2 h of treatment and increased in concentration to ∼0.19 µM after 24 h (Figure 4, *top plots*). When targeted MS was applied to the neuronal side eluate, none of the CPF primary metabolites were observed in any of the neuronal samples (Figure 4, *bottom plots*). Because CPF treatment also corresponded to serum removal from the media, these changes cannot be exclusively attributed to the effects of CPF and more experiments are required to parse the effects of serum versus CPF on barrier integrity. These data along with other studies show TCP to be the primary urinary metabolite of CPF in both humans and rodents (Timchalk et al., 2005; Atabila et al., 2019).

Although the CPF degradation observed in this work agrees with what is seen in humans, the fact that the metabolite concentration decreased tenfold compared to the administered concentration raises questions as to the fate of CPF. One hypothesis is that CPF is removed from the effluent by the PDMS used to fabricate the NVUs. To test PDMS absorption of CPF (Auner et al., 2019), a floating disk experiment was conducted that demonstrated that, after 2 h, 14 ± 1% of the CPF was lost, whereas after 24 h, as much as 67 ± 1.6% of CPF was lost to PDMS (Supplementary Figure S8). These results indicate that the absorption/adsorption of CPF is substantial for PDMS, which has important implications in the design and interpretation of PDMS-based microfluidic experiments. Despite this loss to PDMS, the experiments conducted in this work were run under a continuous flow of 2 μL/min. Under this flow, the CPF-dosed media took less than 9 min to flow through the PDMS-based device. Therefore, uptake of CPF by PDMS only accounts for a small portion of the total CPF removal observed.

If CPF is metabolized within the NVU, where is it going and how can it be tracked? Other *in vitro* BBB studies that have set out to utilize MS to monitor CPF were unable to detect CPF or its metabolites, leading researchers to rely on acetylcholinesterase inhibition instead (Balbuena et al., 2010). Although the CPF metabolites detected in this current work give promise to MS as a tool for tracking organophosphate toxicity, the analysis may need to be expanded beyond the NVU eluate. The increased acetylcholine levels measured on the neuronal side indicate that perhaps CPF, CPO, or other soluble factors were able to cross the BBB but attached to the cells or matrix in the neuronal chamber, allowing them to remain undetected by targeted MS methods. The results encourage additional targeted MS developments to assay the cellular/matrix components for retained CPF.

Alternatively, the low detection of CPF may be due to it being bound to other soluble molecules in the media. CPF plasma protein binding has been measured using varying concentrations of rat and human albumin in buffer (0.04–20 mg/ml) and CPF (0.009 and 0.29 μM). At both of these CPF concentrations, it was reported that when albumin concentrations were high, 99% of the CPF was bound (Lowe et al., 2009). All the experiments presented in this work have either serum or growth factors added to the media that may be binding the CPF and/or CPO in solution. Because CPF is highly bound to proteins *in vivo*, removing the supplement proteins in addition to the serum would lead to higher unbound CPF and greater ability to cross the membrane, but the biological relevance would be compromised (Smith et al., 2012; Smith et al., 2017). Indeed, it has been reported that under physiological conditions CPF membrane transport increases, whereas CPF membrane permeability increases when physiological conditions are removed. In these reports, serum protein concentration was the most substantial factor affecting this transition (Smith et al., 2012). Acid hydrolysis has also been used to recover CPF lost to conjugation, doubling CPF recovery in some cases (Nolan et al., 1984). CPF recovery has also been increased by treating with glucuronidase, liberating TCP glucuronide conjugates (Nolan et al., 1984). These insights into tracking CPF in eluate encourage future studies that include additional processing steps to increase CPF recovery or MS studies which implement broad, untargeted MS assays to assess wide-scale unanticipated metabolic changes (Balbuena et al., 2010; May et al., 2015; May et al., 2016; Sherrod and McLean, 2016; Huang et al., 2019).

## Conclusion

This study demonstrates the potential of the NVU and the power of electrochemistry and MS as a predictive platform for organophosphate toxicology. CPF’s role in society at the intersection of food security and environmental policy in addition to its potential hazard to human health makes understanding its effects critical. When environmental exposure was simulated by challenging the vascular side of the NVU with CPF, neither the pesticide nor any of its metabolites were observed to cross the engineered BBB until the CPF concentration rose to 30 µM. Interestingly, neither CPF nor its active oxon form, CPO, was detected in any of the samples, whereas the inactive metabolites, TCP and DETP, were detected. Although CPF metabolism was observed, a majority of CPF was unaccounted for, suggesting that the analysis may need to be expanded past the NVU eluate. Regardless of the fate of CPF, the treatments were found to cause significant disruption of acetylcholine metabolism at and across the BBB, providing chemical evidence of the substantial disruption induced by this widely used commercial pesticide. These results support previous studies showing that organotypic cultures and their respective analytical platforms enable the identification of primary and secondary mechanisms of action across the BBB. These data validate the predictive power of the NVU and the utility of electrochemistry and MS in identifying chemical exposure events while providing chemical evidence of the substantial disruption in acetylcholine metabolism induced by this widely used commercial pesticide.

## Data Availability

The original contributions presented in the study are included in the article/Supplementary Material; further inquiries can be directed to the corresponding author.

## References

[B1] Adriani G. Ma D. Pavesi A. Kamm R. D. Goh E. L. K. (2017). A 3D Neurovascular Microfluidic Model Consisting of Neurons, Astrocytes and Cerebral Endothelial Cells As a Blood-Brain Barrier. Lab. Chip 17 (3), 448–459. 10.1039/c6lc00638h 28001148

[B2] Allt G. Lawrenson J. G. (2001). Pericytes: Cell Biology and Pathology. Cells Tissues Organs 169, 1–11. 10.1159/000047855 11340256

[B3] Amin H. Maccione A. Marinaro F. Zordan S. Nieus T. Berdondini L. (2016). Electrical Responses and Spontaneous Activity of Human IPS-Derived Neuronal Networks Characterized for 3-Month Culture with 4096-Electrode Arrays. Front. Neurosci. 10, 121. 10.3389/fnins.2016.00121 27065786 PMC4811967

[B4] Andersen M. E. (2014). Developing Microphysiological Systems for Use as Regulatory Tools - Challenges and Opportunities. ALTEX 31 (3), 364–367. 10.14573/altex.1405151 25061900 PMC4778746

[B5] Araci I. E. Quake S. R. (2012). Microfluidic Very Large Scale Integration (MVLSI) with Integrated Micromechanical Valves. Lab. Chip 12 (16), 2803–2806. 10.1039/c2lc40258k 22714259

[B6] Atabila A. Phung D. T. Sadler R. Connell D. Chu C. (2019). Comparative Evaluation of Chlorpyrifos Exposure Estimates from Whole-Body Dermal Dosimetry and Urinary Trichloro-2-Pyridinol (TCP) Methods. Ecotoxicology Environ. Saf. 172, 439–443. 10.1016/J.ECOENV.2019.01.077 30735976

[B7] Auner A. W. Tasneem K. M. Markov D. A. McCawley L. J. Hutson M. S. (2019). Chemical-PDMS Binding Kinetics and Implications for Bioavailability in Microfluidic Devices. Lab. Chip 19 (5), 864–874. 10.1039/c8lc00796a 30720811 PMC6512955

[B8] Balaji V. Castro K. Folch A. (2018). A Laser-Engraving Technique for Portable Micropneumatic Oscillators. Micromachines 9 (9), 426. 10.3390/mi9090426 PMC6187360 30424359

[B9] Balbuena P. Li W. Magnin-Bissel G. Meldrum J. B. Ehrich M. (2010). Comparison of Two Blood-Brain Barrier *In Vitro* Systems: Cytotoxicity and Transfer Assessments of Malathion/Oxon and Lead Acetate. Toxicol. Sci. 114 (2), 260–271. 10.1093/toxsci/kfq001 20064834

[B10] Banerjee J. Shi Y. Azevedo H. S. (2016). *In Vitro* blood-brain Barrier Models for Drug Research: State-Of-The-Art and New Perspectives on Reconstituting These Models on Artificial Basement Membrane Platforms. Drug Discov. Today 21, 1367–1386. Elsevier Ltd September 1. 10.1016/j.drudis.2016.05.020 27283274

[B11] Bang S. Lee S.-R. Ko J. Son K. Tahk D. Ahn J. (2017). A Low Permeability Microfluidic Blood-Brain Barrier Platform with Direct Contact between Perfusable Vascular Network and Astrocytes. Sci. Rep. 7 (1), 1–10. 10.1038/s41598-017-07416-0 28808270 PMC5556097

[B12] Booth R. Kim H. (2012). Characterization of a Microfluidic *In Vitro* Model of the Blood-Brain Barrier (μBBB). Lab. Chip 12 (10), 1784–1792. 10.1039/c2lc40094d 22422217

[B13] Brown J. A. Codreanu S. G. Shi M. Sherrod S. D. Markov D. A. Neely M. D. (2016). Metabolic Consequences of Inflammatory Disruption of the Blood-Brain Barrier in an Organ-On-Chip Model of the Human Neurovascular Unit. J. Neuroinflammation 13 (1), 306. 10.1186/s12974-016-0760-y 27955696 PMC5153753

[B14] Brown J. A. Pensabene V. Markov D. A. Allwardt V. Neely M. D. Shi M. (2015). Recreating Blood-Brain Barrier Physiology and Structure on Chip: A Novel Neurovascular Microfluidic Bioreactor. Biomicrofluidics 9 (5), 054124. 10.1063/1.4934713 26576206 PMC4627929

[B15] Bruner-Tran K. L. Gnecco J. Ding T. Glore D. R. Pensabene V. Osteen K. G. (2017). Exposure to the Environmental Endocrine Disruptor TCDD and Human Reproductive Dysfunction: Translating Lessons from Murine Models. Reprod. Toxicol. 68, 59–71. 10.1016/j.reprotox.2016.07.007 27423904 PMC5237424

[B16] Campisi M. Shin Y. Osaki T. Hajal C. Chiono V. Kamm R. D. (2018). 3D Self-Organized Microvascular Model of the Human Blood-Brain Barrier with Endothelial Cells, Pericytes and Astrocytes. Biomaterials 180, 117–129. 10.1016/j.biomaterials.2018.07.014 30032046 PMC6201194

[B17] Carlson K. Jortner B. S. Ehrich M. (2000). Organophosphorus Compound-Induced Apoptosis in SH-Sy5y Human Neuroblastoma Cells. Toxicol. Appl. Pharmacol. 168 (2), 102–113. 10.1006/taap.2000.8997 11032765

[B18] National Pesticide Information Center (2019). Chlorpyrifos Technical Fact Sheet. Available at: http://npic.orst.edu/factsheets/archive/chlorptech.html (Accessed Dec 18, 2019).

[B19] Cucullo L. Marchi N. Hossain M. Janigro D. (2011). A Dynamic *In Vitro* BBB Model for the Study of Immune Cell Trafficking into the Central Nervous System. J. Cereb. Blood Flow Metab. 31 (2), 767–777. 10.1038/jcbfm.2010.162 20842162 PMC3049530

[B20] Daneman R. Prat A. (2015). The Blood-Brain Barrier. Cold Spring Harb. Perspect. Biol. 7 (1), a020412. 10.1101/cshperspect.a020412 25561720 PMC4292164

[B21] Dauchy S. Dutheil F. Weaver R. J. Chassoux F. Daumas-Duport C. Couraud P.-O. (2008). ABC Transporters, Cytochromes P450 and Their Main Transcription Factors: Expression at the Human Blood-Brain Barrier. J. Neurochem. 107 (6), 1518–1528. 10.1111/j.1471-4159.2008.05720.x 19094056

[B22] DiMasi J. A. Grabowski H. G. Hansen R. W. (2016). Innovation in the Pharmaceutical Industry: New Estimates of R&D Costs. J. Health Econ. 47, 20–33. 10.1016/j.jhealeco.2016.01.012 26928437

[B23] Dingle Y.-T. L. Boutin M. E. Chirila A. M. Livi L. L. Labriola N. R. Jakubek L. M. (2015). Three-Dimensional Neural Spheroid Culture: AnIn VitroModel for Cortical Studies. Tissue Eng. C: Methods 21 (12), 1274–1283. 10.1089/ten.tec.2015.0135 PMC4663656 26414693

[B24] Fennema E. Rivron N. Rouwkema J. Van Blitterswijk C. De Boer J. (2013). Spheroid Culture as a Tool for Creating 3D Complex Tissues. Trends Biotechnol. 31, 108–115. 10.1016/j.tibtech.2012.12.003 23336996

[B25] Fernandes J. T. S. Chutna O. Chu V. Conde J. P. Outeiro T. F. (2016). A Novel Microfluidic Cell Co-culture Platform for the Study of the Molecular Mechanisms of Parkinson's Disease and Other Synucleinopathies. Front. Neurosci. 10, 511. 10.3389/fnins.2016.00511 27895548 PMC5108800

[B26] Ghosh C. Gonzalez-Martinez J. Hossain M. Cucullo L. Fazio V. Janigro D. (2010). Pattern of P450 Expression at the Human Blood-Brain Barrier: Roles of Epileptic Condition and Laminar Flow. Epilepsia 51 (8), 1408–1417. 10.1111/j.1528-1167.2009.02428.x 20074231 PMC3386640

[B27] Grebenyuk S. Ranga A. (2019). Engineering Organoid Vascularization. Front. Bioeng. Biotechnol. 7, 39. 10.3389/fbioe.2019.00039 30941347 PMC6433749

[B28] Griep L. M. Wolbers F. de Wagenaar B. ter Braak P. M. Weksler B. B. Romero I. A. (2013). BBB on CHIP: Microfluidic Platform to Mechanically and Biochemically Modulate Blood-Brain Barrier Function. Biomed. Microdevices 15 (1), 145–150. 10.1007/s10544-012-9699-7 22955726

[B29] Hamilton N. B. (2010). Pericyte-Mediated Regulation of Capillary Diameter: A Component of Neurovascular Coupling in Health and Disease. Front. Neuroenerg. 2, 5. 10.3389/fnene.2010.00005 PMC2912025 20725515

[B30] Hasan M. Berdichevsky Y. (2016). Neural Circuits on a Chip. Micromachines 7, 157. 10.3390/mi7090157 PMC6190100 30404330

[B31] Helms H. C. Abbott N. J. Burek M. Cecchelli R. Couraud P.-O. Deli M. A. (2016). *In Vitro* models of the Blood-Brain Barrier: An Overview of Commonly Used Brain Endothelial Cell Culture Models and Guidelines for Their Use. J. Cereb. Blood Flow Metab. 36 (5), 862–890. 10.1177/0271678X16630991 26868179 PMC4853841

[B32] Helms H. C. Brodin B. (2014). Generation of Primary Cultures of Bovine Brain Endothelial Cells and Setup of Cocultures with Rat Astrocytes. Methods Mol. Biol. 1135, 365–382. 10.1007/978-1-4939-0320-7_30 24510879

[B33] Herculano-Houzel S. (2009). The Human Brain in Numbers: A Linearly Scaled-Up Primate Brain. Front. Hum. Neurosci. 3, 31. 10.3389/neuro.09.031.2009 19915731 PMC2776484

[B34] Hopkins A. M. DeSimone E. Chwalek K. Kaplan D. L. (2015). 3D *In Vitro* Modeling of the Central Nervous System. Prog. Neurobiol. 125, 1–25. Elsevier Ltd February 1. 10.1016/j.pneurobio.2014.11.003 25461688 PMC4324093

[B35] Huang R. Grishagin I. Wang Y. Zhao T. Greene J. Obenauer J. C. (2019). The NCATS BioPlanet - an Integrated Platform for Exploring the Universe of Cellular Signaling Pathways for Toxicology, Systems Biology, and Chemical Genomics. Front. Pharmacol. 10, 445. 10.3389/fphar.2019.00445 31133849 PMC6524730

[B36] Hutson M. S. Alexander P. G. Allwardt V. Aronoff D. M. Bruner-Tran K. L. Cliffel D. E. (2016). Organs-on-Chips as Bridges for Predictive Toxicology. Appl. Vitro Toxicol. 2 (2), 97–102. 10.1089/aivt.2016.0003

[B37] Jeong G. S. Chang J. Y. Park J. S. Lee S.-A. Park D. Woo J. (2015). Networked Neural Spheroid by Neuro-Bundle Mimicking Nervous System Created by Topology Effect. Mol. Brain 8, 17. 10.1186/s13041-015-0109-y 25888468 PMC4379946

[B38] Karanth S. Liu J. Mirajkar N. Pope C. (2006). Effects of Acute Chlorpyrifos Exposure on *In Vivo* Acetylcholine Accumulation in Rat Striatum. Toxicol. Appl. Pharmacol. 216 (1), 150–156. 10.1016/J.TAAP.2006.04.006 16777161

[B39] Kashyap M. P. Singh A. K. Kumar V. Tripathi V. K. Srivastava R. K. Agrawal M. (2011). Monocrotophos Induced Apoptosis in PC12 Cells: Role of Xenobiotic Metabolizing Cytochrome P450S. PLoS One 6 (3), e17757. 10.1371/journal.pone.0017757 21445290 PMC3061860

[B40] Kashyap M. P. Singh A. K. Kumar V. Yadav D. K. Khan F. Jahan S. (2013). Pkb/Akt1 Mediates Wnt/GSK3β/β-Catenin Signaling-Induced Apoptosis in Human Cord Blood Stem Cells Exposed to Organophosphate Pesticide Monocrotophos. Stem Cell Develop. 22 (2), 224–238. 10.1089/scd.2012.0220 PMC3545351 22897592

[B41] Kashyap M. P. Singh A. K. Siddiqui M. A. Kumar V. Tripathi V. K. Khanna V. K. (2010). Caspase Cascade Regulated Mitochondria Mediated Apoptosis in Monocrotophos Exposed PC12 Cells. Chem. Res. Toxicol. 23 (11), 1663–1672. 10.1021/tx100234m 20957986

[B42] Kilic O. Pamies D. Lavell E. Schiapparelli P. Feng Y. Hartung T. (2016). Brain-on-a-Chip Model Enables Analysis of Human Neuronal Differentiation and Chemotaxis. Lab. Chip 16 (21), 4152–4162. 10.1039/c6lc00946h 27722368

[B43] Koo Y. Hawkins B. T. Yun Y. (2018). Three-Dimensional (3D) Tetra-Culture Brain on Chip Platform for Organophosphate Toxicity Screening. Sci. Rep. 8 (1), 1–7. 10.1038/s41598-018-20876-2 29434277 PMC5809488

[B44] Lancaster M. A. Renner M. Martin C.-A. Wenzel D. Bicknell L. S. Hurles M. E. (2013). Cerebral Organoids Model Human Brain Development and Microcephaly. Nature 501 (7467), 373–379. 10.1038/nature12517 23995685 PMC3817409

[B45] Levin E. D. Addy N. Nakajima A. Christopher N. C. Seidler F. J. Slotkin T. A. (2001). Persistent Behavioral Consequences of Neonatal Chlorpyrifos Exposure in Rats. Develop. Brain Res. 130 (1), 83–89. 10.1016/S0165-3806(01)00215-2 11557096

[B46] Li W. Ehrich M. (2013). Transient Alterations of the Blood-Brain Barrier Tight Junction and Receptor Potential Channel Gene Expression by Chlorpyrifos. J. Appl. Toxicol. 33 (10), 1187–1191. 10.1002/jat.2762 22611033

[B47] Liu L. Koo Y. Russell T. Gay E. Li Y. Yun Y. (2020). Three-dimensional Brain-On-Chip Model Using Human iPSC-Derived GABAergic Neurons and Astrocytes: Butyrylcholinesterase post-treatment for Acute Malathion Exposure. PLoS One 15 (3), e0230335. 10.1371/journal.pone.0230335 32163499 PMC7067464

[B48] Lizama B. N. Palubinsky A. M. Raveendran V. A. Moore A. M. Federspiel J. D. Codreanu S. G. (2018). Neuronal Preconditioning Requires the Mitophagic Activity of C-Terminus of HSC70-Interacting Protein. J. Neurosci. 38 (31), 6825–6840. 10.1523/JNEUROSCI.0699-18.2018 29934347 PMC6070662

[B49] Low L. A. Mummery C. Berridge B. R. Austin C. P. Tagle D. A. (2021). Organs-on-Chips: Into the Next Decade. Nat. Rev. Drug Discovnature Res. May 20, 345–361. 10.1038/s41573-020-0079-3 32913334

[B50] Lowe E. R. Poet T. S. Rick D. L. Marty M. S. Mattsson J. L. Timchalk C. (2009). The Effect of Plasma Lipids on the Pharmacokinetics of Chlorpyrifos and the Impact on Interpretation of Blood Biomonitoring Data. Toxicol. Sci. 108 (2), 258–272. 10.1093/toxsci/kfp034 19223661

[B51] Lozano R. Stevens L. Thompson B. C. Gilmore K. J. Gorkin R. Stewart E. M. (2015). 3D Printing of Layered Brain-like Structures Using Peptide Modified Gellan Gum Substrates. Biomaterials 67, 264–273. 10.1016/j.biomaterials.2015.07.022; in 26231917

[B52] Maoz B. M. Herland A. FitzGerald E. A. Grevesse T. Vidoudez C. Pacheco A. R. (2018). A Linked Organ-On-Chip Model of the Human Neurovascular Unit Reveals the Metabolic Coupling of Endothelial and Neuronal Cells. Nat. Biotechnol. 36 (9), 865–874. 10.1038/nbt.4226 30125269 PMC9254231

[B53] Marín-Padilla M. (2012). The Human Brain Intracerebral Microvascular System: Development and Structure. Front. Neuroanat. 6, 1–14. 10.3389/fnana.2012.00038 22993505 PMC3440694

[B54] May J. C. Gant-Branum R. L. McLean J. A. (2016). Targeting the Untargeted in Molecular Phenomics with Structurally-Selective Ion Mobility-Mass Spectrometry. Curr. Opin. Biotechnol. 39, 192–197. 10.1016/j.copbio.2016.04.013 27132126 PMC4899109

[B55] May J. C. Goodwin C. R. McLean J. A. (2015). Ion Mobility-Mass Spectrometry Strategies for Untargeted Systems, Synthetic, and Chemical Biology. Curr. Opin. Biotechnol. 31, 117–121. 10.1016/j.copbio.2014.10.012 25462629 PMC4297680

[B56] McClain E. S. Miller D. R. Cliffel D. E. (2019). Communication—Microfluidic Electrochemical Acetylcholine Detection in the Presence of Chlorpyrifos. J. Electrochem. Soc. 166 (16), 178–181. 10.1149/2.0711916jes

[B57] McCracken K. W. Catá E. M. Crawford C. M. Sinagoga K. L. Schumacher M. Rockich B. E. (2014). Modelling Human Development and Disease in Pluripotent Stem-Cell-Derived Gastric Organoids. Nature 516 (7531), 400–404. 10.1038/nature13863 25363776 PMC4270898

[B58] McKenzie J. R. Cognata A. C. Davis A. N. Wikswo J. P. Cliffel D. E. (2015). Real-Time Monitoring of Cellular Bioenergetics with a Multianalyte Screen-Printed Electrode. Anal. Chem. 87 (15), 7857–7864. 10.1021/acs.analchem.5b01533 26125545 PMC4770793

[B59] McKenzie J. R. Palubinsky A. M. Brown J. E. McLaughlin B. Cliffel D. E. (2012). Metabolic Multianalyte Microphysiometry Reveals Extracellular Acidosis Is an Essential Mediator of Neuronal Preconditioning. ACS Chem. Neurosci. 3 (7), 510–518. 10.1021/cn300003r 22860220 PMC3399578

[B60] Middlemore-Risher M.-L. Adam B.-L. Lambert N. A. Terry A. V. (2011). Effects of Chlorpyrifos and Chlorpyrifos-Oxon on the Dynamics and Movement of Mitochondria in Rat Cortical Neurons. J. Pharmacol. Exp. Ther. 339 (2), 341–349. 10.1124/jpet.111.184762 21799050 PMC3199992

[B61] Mileson B. E. Chambers J. E. Chen W. L. Dettbarn W. Ehrich M. Eldefrawi A. T. (1998). Common Mechanism of Toxicity: A Case Study of Organophosphorus Pesticides. Toxicol. Sci. 41 (1), 8–20. 10.1006/toxs.1997.243110.1093/toxsci/41.1.8 9520337

[B62] Miller D. R. McClain E. S. Cliffel D. E. (2018). Electrochemical Microphysiometry Detects Cellular Glutamate Uptake. J. Electrochem. Soc. 165 (12), G3120–G3124. 10.1149/2.0201812jes

[B63] Mohammed M. I. Haswell S. Gibson I. (2015). Lab-On-A-Chip or Chip-In-A-Lab: Challenges of Commercialization Lost in Translation. Proced. Technol. 20, 54–59. 10.1016/J.PROTCY.2015.07.010

[B64] Moore P. D. Yedjou C. G. Tchounwou P. B. (2010). Malathion-Induced Oxidative Stress, Cytotoxicity, and Genotoxicity in Human Liver Carcinoma (HepG2) Cells. Environ. Toxicol. 25 (3), 221–226. 10.1002/tox.20492 19399848 PMC2862833

[B65] Moreno C. L. Della Guardia L. Shnyder V. Ortiz-Virumbrales M. Kruglikov I. Zhang B. (2018). iPSC-derived Familial Alzheimer's PSEN2 N141I Cholinergic Neurons Exhibit Mutation-dependent Molecular Pathology Corrected by Insulin Signaling. Mol. Neurodegeneration 13 (1), 33. 10.1186/s13024-018-0265-5 PMC6020427 29945658

[B66] Nolan R. J. Rick D. L. Freshour N. L. Saunders J. H. (1984). Chlorpyrifos: Pharmacokinetics in Human Volunteers. Toxicol. Appl. Pharmacol. 73 (1), 8–15. 10.1016/0041-008X(84)90046-2 6200956

[B67] Ortiz-Virumbrales M. Moreno C. L. Kruglikov I. Marazuela P. Sproul A. Jacob S. (2017). CRISPR/Cas9-Correctable Mutation-Related Molecular and Physiological Phenotypes in iPSC-Derived Alzheimer's PSEN2 N141I Neurons. Acta Neuropathol. Commun. 5 (1). 10.1186/s40478-017-0475-z PMC5660456 29078805

[B68] Park J. H. Ko J. Hwang J. Koh H. C. (2015a). Dynamin-Related Protein 1 Mediates Mitochondria-Dependent Apoptosis in Chlorpyrifos-Treated SH-Sy5y Cells. Neurotoxicology 51, 145–157. 10.1016/j.neuro.2015.10.008 26598294

[B69] Park J. Lee B. K. Jeong G. S. Hyun J. K. Lee C. J. Lee S.-H. (2015b). Three-dimensional Brain-On-A-Chip with an Interstitial Level of Flow and its Application as an *In Vitro* Model of Alzheimer's Disease. Lab. Chip 15 (1), 141–150. 10.1039/c4lc00962b 25317977

[B70] Parran D. K. Magnin G. Li W. Jortner B. S. Ehrich M. (2005). Chlorpyrifos Alters Functional Integrity and Structure of an *In Vitro* BBB Model: Co-Cultures of Bovine Endothelial Cells and Neonatal Rat Astrocytes. Neurotoxicology 26 (1), 77–88. 10.1016/j.neuro.2004.07.003 15527875

[B71] Pas S. P. (2018). The Rise of Three-Dimensional Human Brain Cultures. Nature 24, 437–445. 10.1038/nature25032 29364288

[B72] Paşca A. M. Sloan S. A. Clarke L. E. Tian Y. Makinson C. D. Huber N. (2015). Functional Cortical Neurons and Astrocytes from Human Pluripotent Stem Cells in 3D Culture. Nat. Methods 12 (7), 671–678. 10.1038/nmeth.3415 26005811 PMC4489980

[B73] Phan D. T. Bender R. H. F. Andrejecsk J. W. Sobrino A. Hachey S. J. George S. C. (2017). Blood-brain Barrier-On-A-Chip: Microphysiological Systems that Capture the Complexity of the Blood-central Nervous System Interface. Exp. Biol. Med. (Maywood) 242 (17), 1669–1678. 10.1177/1535370217694100 28195514 PMC5786363

[B74] Pimentel E. Sivalingam K. Doke M. Samikkannu T. (2020). Effects of Drugs of Abuse on the Blood-Brain Barrier: A Brief Overview. Front. Neurosci. 14, 513. 10.3389/fnins.2020.00513 32670001 PMC7326150

[B75] Prabhakarpandian B. Shen M.-C. Nichols J. B. Mills I. R. Sidoryk-Wegrzynowicz M. Aschner M. (2013). SyM-BBB: A Microfluidic Blood Brain Barrier Model. Lab. Chip 13 (6), 1093. 10.1039/c2lc41208j 23344641 PMC3613157

[B76] Prendergast M. A. Terry A. V. Buccafusco J. J. (1998). Effects of Chronic, Low-Level Organophosphate Exposure on Delayed Recall, Discrimination, and Spatial Learning in Monkeys and Rats. Neurotoxicology and Teratology 20 (2), 115–122. 10.1016/S0892-0362(97)00098-6 9536457

[B77] Pridgeon C. S. Schlott C. Wong M. W. Heringa M. B. Heckel T. Leedale J. (2018). Innovative Organotypic *In Vitro* Models for Safety Assessment: Aligning with Regulatory Requirements and Understanding Models of the Heart, Skin, and Liver as Paradigms. Arch. Toxicol. 92, 557–569. 10.1007/s00204-018-2152-9 29362863 PMC5818581

[B78] Raimondi I. Izzo L. Tunesi M. Comar M. Albani D. Giordano C. (2020). Organ-On-A-Chip *In Vitro* Models of the Brain and the Blood-Brain Barrier and Their Value to Study the Microbiota-Gut-Brain Axis in Neurodegeneration. Front. Bioeng. Biotechnol. 7, 435. 10.3389/fbioe.2019.00435 31998702 PMC6965718

[B79] Rauh V. A. Perera F. P. Horton M. K. Whyatt R. M. Bansal R. Hao X. (2012). Brain Anomalies in Children Exposed Prenatally to a Common Organophosphate Pesticide. Proc. Natl. Acad. Sci. 109 (20), 7871–7876. 10.1073/pnas.1203396109 22547821 PMC3356641

[B80] Rauh V. Arunajadai S. Horton M. Perera F. Hoepner L. Barr D. B. (2011). Seven-Year Neurodevelopmental Scores and Prenatal Exposure to Chlorpyrifos, a Common Agricultural Pesticide. Environ. Health Perspect. 119 (8), 1196–1201. 10.1289/ehp.1003160 21507777 PMC3237355

[B81] Saulsbury M. D. Heyliger S. O. Wang K. Johnson D. J. (2009). Chlorpyrifos Induces Oxidative Stress in Oligodendrocyte Progenitor Cells. Toxicology 259 (1–2), 1–9. 10.1016/J.TOX.2008.12.026 19167454

[B82] Schadt E. E. Buchanan S. Brennand K. J. Merchant K. M. (2014). Evolving toward a Human-Cell Based and Multiscale Approach to Drug Discovery for CNS Disorders. Front. Pharmacol. 5, 252. 10.3389/fphar.2014.00252 25520658 PMC4251289

[B83] Shamir E. R. Ewald A. J. (2014). Three-Dimensional Organotypic Culture: Experimental Models of Mammalian Biology and Disease. Nat. Rev. Mol. Cel Biol 15, 647–664. Nature Publishing Group January. 10.1038/nrm3873 PMC4352326 25237826

[B84] Sherrod S. D. McLean J. A. (2016). Systems-Wide High-Dimensional Data Acquisition and Informatics Using Structural Mass Spectrometry Strategies. Clin. Chem. 62 (1), 77–83. 10.1373/clinchem.2015.238261 26453699 PMC4843518

[B85] Slotkin T. A. (2011). Does Early-Life Exposure to Organophosphate Insecticides Lead to Prediabetes and Obesity? Reprod. Toxicol. 31 (3), 297–301. 10.1016/j.reprotox.2010.07.012 20850519 PMC3025269

[B86] Smith J. N. Carver Z. A. Weber T. J. Timchalk C. (2017). Predicting Transport of 3,5,6-Trichloro-2-Pyridinol into Saliva Using a Combination Experimental and Computational Approach. Toxicol. Sci. 157 (2), 438–450. 10.1093/toxsci/kfx055 28402492

[B87] Smith J. N. Hinderliter P. M. Timchalk C. Bartels M. J. Poet T. S. (2014). A Human Life-Stage Physiologically Based Pharmacokinetic and Pharmacodynamic Model for Chlorpyrifos: Development and Validation. Regul. Toxicol. Pharmacol. 69 (3), 580–597. 10.1016/j.yrtph.2013.10.005 24200834

[B88] Smith J. N. Wang J. Lin Y. Klohe E. M. Timchalk C. (2012). Pharmacokinetics and Pharmacodynamics of Chlorpyrifos and 3,5,6-Trichloro-2-Pyridinol in Rat Saliva after Chlorpyrifos Administration. Toxicol. Sci. 130 (2), 245–256. 10.1093/toxsci/kfs251 22874420

[B89] Soscia D. Belle A. Fischer N. Enright H. Sales A. Osburn J. (2017). Controlled Placement of Multiple CNS Cell Populations to Create Complex Neuronal Cultures. PLoS One 12 (11), e0188146. 10.1371/journal.pone.0188146 29161298 PMC5697820

[B90] Sultatos L. G. (1994). Mammalian Toxicology of Organophosphorus Pesticides. J. Toxicol. Environ. Health 43 (3), 271–289. 10.1080/15287399409531921 7966438

[B91] Takayama S. McDonald J. C. Ostuni E. Liang M. N. Kenis P. J. A. Ismagilov R. F. (1999). Patterning Cells and Their Environments Using Multiple Laminar Fluid Flows in Capillary Networks. Proc. Natl. Acad. Sci. 96 (10), 5545–5548. 10.1073/pnas.96.10.5545 10318920 PMC21896

[B92] Tang J. Rose R. L. Chambers J. E. (2006). “Metabolism of Organophosphorus and Carbamate Pesticides,” in Toxicology of Organophosphate & Carbamate Compounds (Redmond, Washington: Elsevier), 127–143. 10.1016/B978-012088523-7/50011-9

[B93] Tang-Schomer M. D. White J. D. Tien L. W. Schmitt L. I. Valentin T. M. Graziano D. J. (2014). Bioengineered Functional Brain-like Cortical Tissue. Proc. Natl. Acad. Sci. 111 (38), 13811–13816. 10.1073/pnas.1324214111 25114234 PMC4183301

[B94] Taylor P. Brown J. (1999). Basic Neurochemistry: Synthesis, Storage and Release of Acetylcholine, 6th ed. Editor-in-Chief Siegel G J. Agranoff B. W. Albers R. W. Fisher S. K. Uhler M. D.. Philidelphia, PA: Lippincott-Raven.

[B95] Theobald J. Ghanem A. Wallisch P. Banaeiyan A. A. Andrade-Navarro M. A. Taškova K. (2018). Liver-Kidney-on-Chip to Study Toxicity of Drug Metabolites. ACS Biomater. Sci. Eng. 4 (1), 78–89. 10.1021/acsbiomaterials.7b00417 33418680

[B96] Timchalk C. Poet T. S. Hinman M. N. Busby A. L. Kousba A. A. (2005). Pharmacokinetic and Pharmacodynamic Interaction for a Binary Mixture of Chlorpyrifos and Diazinon in the Rat. Toxicol. Appl. Pharmacol. 205 (1), 31–42. 10.1016/J.TAAP.2004.09.004 15885262

[B97] Vernetti L. Gough A. Baetz N. Blutt S. Broughman J. R. Brown J. A. (2017). Functional Coupling of Human Microphysiology Systems: Intestine, Liver, Kidney Proximal Tubule, Blood-Brain Barrier and Skeletal Muscle. Sci. Rep. 7, 42296. 10.1038/srep42296 28176881 PMC5296733

[B98] Volpatti L. R. Yetisen A. K. (2014). Commercialization of Microfluidic Devices. Trends Biotechnol. 32 (7), 347–350. 10.1016/J.TIBTECH.2014.04.010 24954000

[B99] Voorhees J. R. Rohlman D. S. Lein P. J. Pieper A. A. (2017). Neurotoxicity in Preclinical Models of Occupational Exposure to Organophosphorus Compounds. Front. Neurosci. 10, 590. 10.3389/fnins.2016.00590 28149268 PMC5241311

[B100] Wang Y. I. Abaci H. E. Shuler M. L. (2017). Microfluidic Blood-Brain Barrier Model Provides In Vivo‐like Barrier Properties for Drug Permeability Screening. Biotechnol. Bioeng. 114 (1), 184–194. 10.1002/bit.26045 27399645 PMC6650146

[B101] Wang Y. Wang L. Zhu Y. Qin J. (2018). Human Brain Organoid-On-A-Chip to Model Prenatal Nicotine Exposure. Lab. Chip 18 (6), 851–860. 10.1039/c7lc01084b 29437173

[B102] Yang J. Mutkus L. A. Sumner D. Stevens J. T. Eldridge J. C. Strandhoy J. W. (2001). Transendothelial Permeability of Chlorpyrifos in RBE4 Monolayers Is Modulated by Astrocyte-Conditioned Medium. Mol. Brain Res. 97 (1), 43–50. 10.1016/S0169-328X(01)00296-0 11744161

[B103] Zhang Y. S. Aleman J. Shin S. R. Kilic T. Kim D. Mousavi Shaegh S. A. (2017). Multisensor-Integrated Organs-On-Chips Platform for Automated and Continual *In Situ* Monitoring of Organoid Behaviors. Proc. Natl. Acad. Sci. USA 114 (12), E2293–E2302. 10.1073/pnas.1612906114 28265064 PMC5373350

[B104] Zhuang P. Sun A. X. An J. Chua C. K. Chew S. Y. (2018). 3D Neural Tissue Models: From Spheroids to Bioprinting. Biomaterials 154, 113–133. 10.1016/j.biomaterials.2017.10.002 29120815

