# Supplementary material for: Chlorpyrifos Disrupts Acetylcholine Metabolism Across Model Blood-Brain Barrier
Source: Front Bioeng Biotechnol. 2021 Aug 27;9:622175. doi: 10.3389/fbioe.2021.622175 (PMC8431803; doi:10.3389/fbioe.2021.622175)
Supplement: Supplementary file 1 [file table2.docx]

Frontiers Graphical Abstract


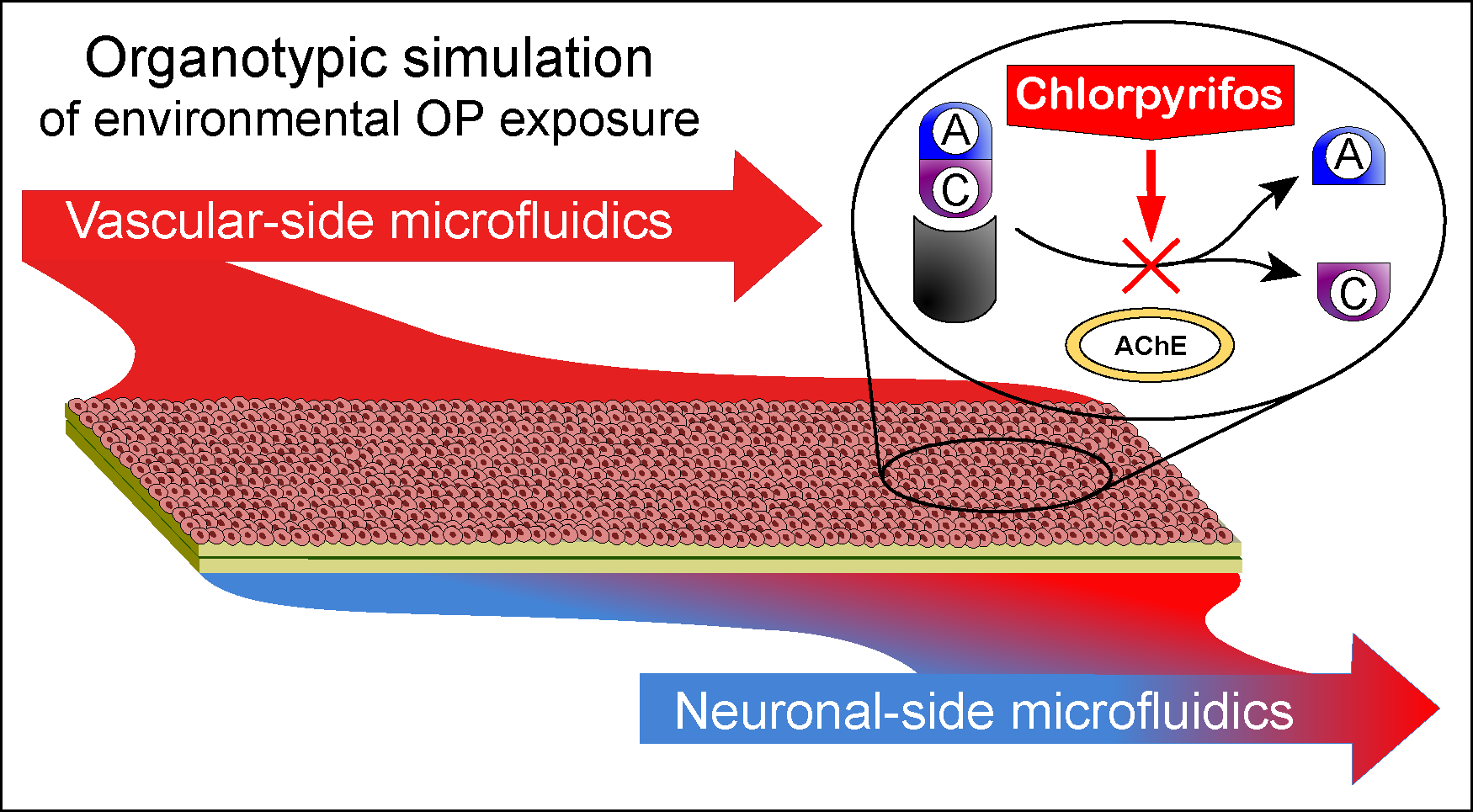


An organotypic blood-brain barrier was used to simulate environmental exposure to a commonly used organophosphate (OP). The substantial disruption observed in this work reinforces what is known about OP metabolism and mechanisms of impact, validates use of the NVUs for OP toxicology testing, and provides a model platform for analyzing these organotypic systems.
