# Supplementary material for: Chlorpyrifos Disrupts Acetylcholine Metabolism Across Model Blood-Brain Barrier
Source: Front Bioeng Biotechnol. 2021 Aug 27;9:622175. doi: 10.3389/fbioe.2021.622175 (PMC8431803; doi:10.3389/fbioe.2021.622175)
Supplement: Supplementary file 2 [file DataSheet1.pdf]

Supplemental information.

Analyte	Mass Transition (Da)	Retention Time (min)	Ion Polarity
Chlorpyrifos (CPF)	350.0 → 97.0	2.54	Positive
Chlorpyrifos-oxon (CPO)	334.0 → 277.9	1.22	Positive
Diethylthiophosphate (DETP)	169.0 → 95.0	0.49	Negative
Trichloropyridinol (TCP)	192.2 → 176.1	2.94	Positive
Di-*tert*-butylpyridine (DtBP)	195.9 → 195.9	0.86	Negative

Supplementary Table 1. A list of metabolites, mass transitions, retention times, and ion polarities used for the targeted MS analysis. All mass transitions correspond to the protonated $[M+H]^+$ ion form of each analyte.

CPF Dose (µM)	Analyte Concentration Detected (µM)				
	CPF	CPO	DETP	TCP	DtBP (I.S.)
**Vascular Media Samples**					
Control	0.001 ± 0.000	0.001 ± 0.000	0.012 ± 0.008	0.001 ± 0.002	0.530 ± 0.019
0	0.001 ± 0.001	0.000 ± 0.000	0.013 ± 0.004	0.002 ± 0.001	0.498 ± 0.052
1	0.000 ± 0.001	0.000 ± 0.000	0.014 ± 0.004	0.086 ± 0.037	0.501 ± 0.066
3	0.000 ± 0.000	0.000 ± 0.000	0.018 ± 0.010	0.244 ± 0.033	0.494 ± 0.048
10	0.000 ± 0.001	0.000 ± 0.000	0.022 ± 0.006	0.607 ± 0.091	0.498 ± 0.063
30	0.001 ± 0.001	0.000 ± 0.000	0.026 ± 0.006	1.460 ± 0.100	0.502 ± 0.032
100	0.001 ± 0.001	0.000 ± 0.000	0.091 ± 0.006	5.724 ± 0.484	0.507 ± 0.051
**Neuronal Media Samples**					
Control	0.003 ± 0.002	0.000 ± 0.000	0.010 ± 0.006	0.000 ± 0.000	0.521 ± 0.009
0	0.001 ± 0.001	0.000 ± 0.000	0.012 ± 0.011	0.000 ± 0.000	0.527 ± 0.018
1	0.002 ± 0.001	0.000 ± 0.000	0.009 ± 0.004	0.005 ± 0.005	0.517 ± 0.020
3	0.000 ± 0.000	0.000 ± 0.000	0.014 ± 0.008	0.011 ± 0.003	0.475 ± 0.049
10	0.001 ± 0.001	0.000 ± 0.000	0.012 ± 0.002	0.019 ± 0.007	0.485 ± 0.027
30	0.006 ± 0.005	0.000 ± 0.000	0.012 ± 0.002	0.073 ± 0.018	0.499 ± 0.023
100	0.010 ± 0.007	0.000 ± 0.000	0.025 ± 0.009	0.387 ± 0.109	0.497 ± 0.006

Supplementary Table 2. Concentrations of CPF and its metabolites measured from quantitative MS for the vascular (top rows) and neuronal (bottom rows) media samples obtained from the NVU escalating dose experiments. Concentrations represent average values from replicate measurements. The concentrations measured for internal standard (I.S.), DtBP, is also shown. The limit of quantitation (LOQ) was determined to be 0.010 µM, based on the linear response observed from the multi-point TCP calibration.

Collection Time (hr)	Analyte Concentration Detected (µM)				
	CPF	CPO	DETP	TCP	DtBP (I.S.)
**Vascular Media Samples**					
Control	0.000 ± 0.000	0.000 ± 0.000	0.009 ± 0.001	0.000 ± 0.000	0.041 ± 0.013
0	0.000 ± 0.000	0.000 ± 0.000	0.010 ± 0.001	0.001 ± 0.000	0.021 ± 0.010
2	0.000 ± 0.000	0.000 ± 0.000	0.008 ± 0.001	0.059 ± 0.005	0.032 ± 0.008
4	0.000 ± 0.000	0.000 ± 0.000	0.008 ± 0.001	0.084 ± 0.003	0.039 ± 0.010
8	0.000 ± 0.000	0.000 ± 0.000	0.007 ± 0.001	0.110 ± 0.004	0.037 ± 0.001
24	0.000 ± 0.000	0.000 ± 0.000	0.009 ± 0.001	0.192 ± 0.012	0.044 ± 0.007
**Neuronal Media Samples**					
Control	0.000 ± 0.000	0.000 ± 0.000	0.009 ± 0.002	0.000 ± 0.000	0.043 ± 0.011
0	0.000 ± 0.000	0.000 ± 0.000	0.011 ± 0.000	0.000 ± 0.000	0.028 ± 0.003
2	0.000 ± 0.000	0.000 ± 0.000	0.007 ± 0.001	0.000 ± 0.000	0.044 ± 0.005
4	0.000 ± 0.000	0.000 ± 0.000	0.009 ± 0.001	0.000 ± 0.000	0.042 ± 0.004
8	0.000 ± 0.000	0.000 ± 0.000	0.008 ± 0.001	0.000 ± 0.000	0.040 ± 0.002
24	0.001 ± 0.000	0.000 ± 0.000	0.009 ± 0.001	0.000 ± 0.000	0.044 ± 0.008

Supplementary Table 3. Concentrations of CPF and its metabolites measured from quantitative MS for the vascular (top rows) and neuronal (bottom rows) media samples obtained from the NVU time course at constant dose (10 µM CPF) experiments.

Using microphysiometry, cytotoxicity can be explored by tracking metabolic disruption in specific pathways, such as cholinergic signaling and/or metabolism. Since organophosphates can inhibit acetylcholinesterase, an enzyme used in the sensor fabrication, the effect of CPF on the function of the electrochemical acetylcholine sensor was determined before this sensor was used further (**Supplemental Table 2**).[38] Even after a 30-minute exposure to 50 µM CPF, the sensor exhibited negligible signal losses compared to control (5.8 ± 1.4% vs. 7.3 ± 2.8% respectively, $p = 0.62$). It should be noted that, although exposures lasted up to 24 hrs, sample measurement only required two minutes of exposure of the sensor to the sample (and therefore CPF). This low signal interference demonstrates the sensor's utility in measuring acetylcholine levels even in the presence of CPF.

**Acetylcholine sensor parameters**	**25**°C
**Detection limit (**μM)	0.2 ± 0.1
**Quantitation limit (**μM)	0.7 ± 0.1
**Sensitivity (**nA μM^{-1} mm^{-2})	0.9 ± 0.1
**Linear range (**μM)	1-150

Supplementary Table 4. Acetylcholine sensor parameters determined within the μCA electrochemical detection platform. Calibrations were performed by monitoring the current generated by 21 calibrant solutions (5 μM to 5 mM acetylcholine) in buffer (2 mM PBS, 120 mM KCl, pH 7). Calibrants were sampled through a MicroFormulator at a flow rate of 100 μL/min and monitored by a CHI 1440 potentiostat (CH Instruments, Austin, TX) held at 0.6 V vs. Ag/AgCl with buffer in between to provide a baseline (two minutes each). The detection and quantitation limits, along with the sensitivity and linear range of the sensor, were determined by performing a linear regression on the calibration data. The maximum limit of linearity was determined by visual analysis of the calibration curve. The LOD was calculated by multiplying the error of the blank by three (10X for LOQ) and dividing by the slope of the determined linear range.

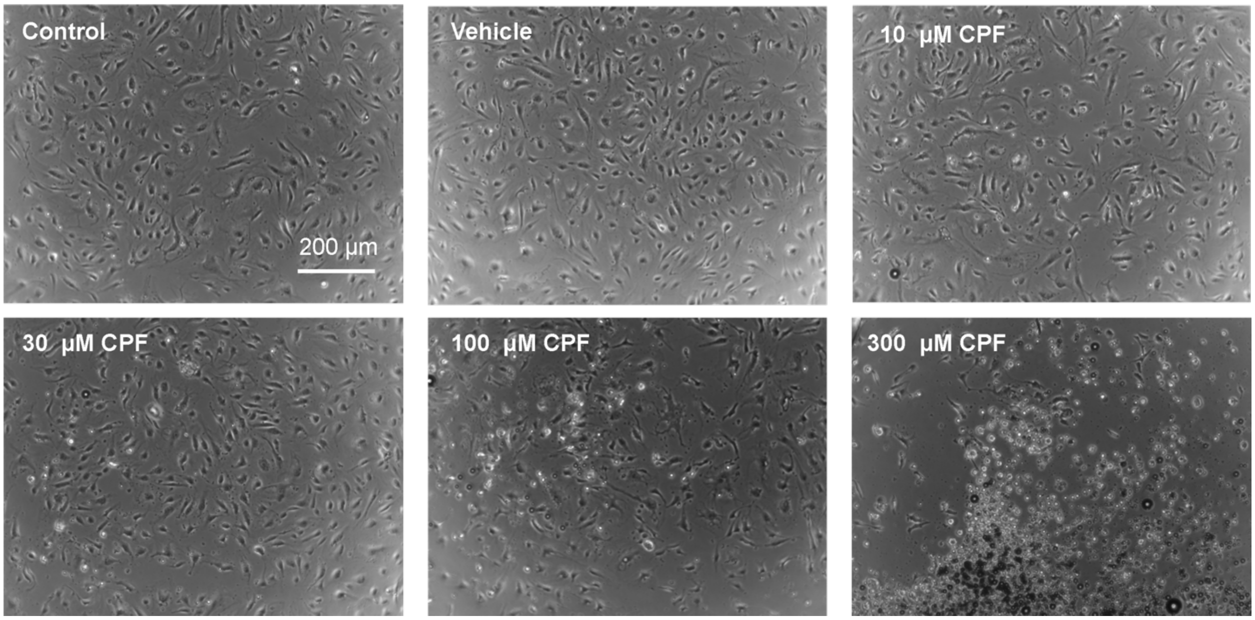

Supplemental Figure 1. hBMVECs response to CPF treatment. hBMVECs plated at 120,000 cells/mL on PLO-coated 24 well plates and grown for 24 hours before treatments. Cells were then treated with either vehicle, media change control, or with varying concentrations of CPF (0, 10, 100, 300 μM CPF). Cells were grown in endothelial basal media 2 (EBM2) containing 5% fetal bovine serum (FBS), growth bullet kit, and 1% penicillin and streptomycin. Experiments were conducted at 37 ºC and 5% CO_2. Images complements of Prof. BethAnn McLaughlin and Amy Palubinksy, Ph.D.

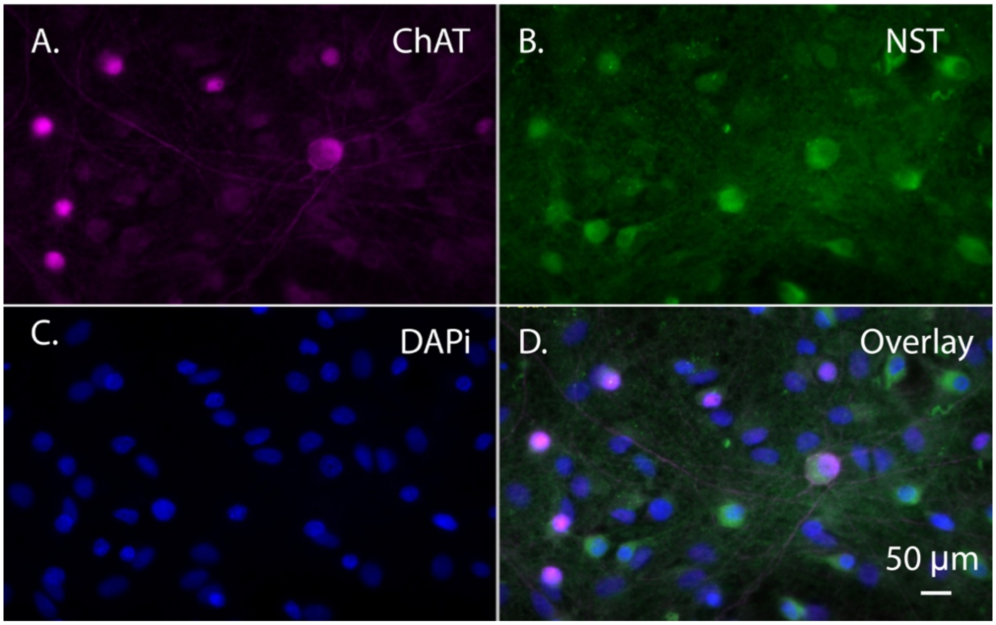

Supplemental Figure 2. Neurochemical staining of cultures revealing a high number of acetylcholine positive cells within our immature neurons. Staining for **A.** choline acetyl transferase (ChAT), **B.** Neuron Specific Tubulin (NST) **C.** 4′,6-diamidino-2-phenylindole (DAPi) and **D.** overlayed showing ~10% cholinergic neurons. Images complements of Prof. BethAnn McLaughlin and Amy Palubinksy, Ph.D.

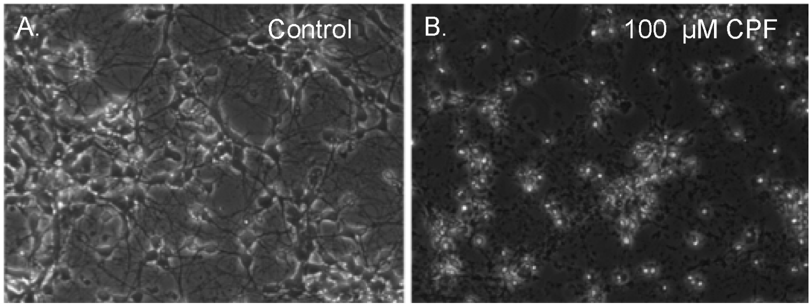

Supplemental Figure 3. Neuronal response to CPF treatment. Neurons were either treated with a media change control or 100 µM CPF for 18 hours and imaged. Experiments were conducted at 37 ºC and 5% CO_2. Images complements of Prof. BethAnn McLaughlin and Amy Palubinksy, Ph.D.

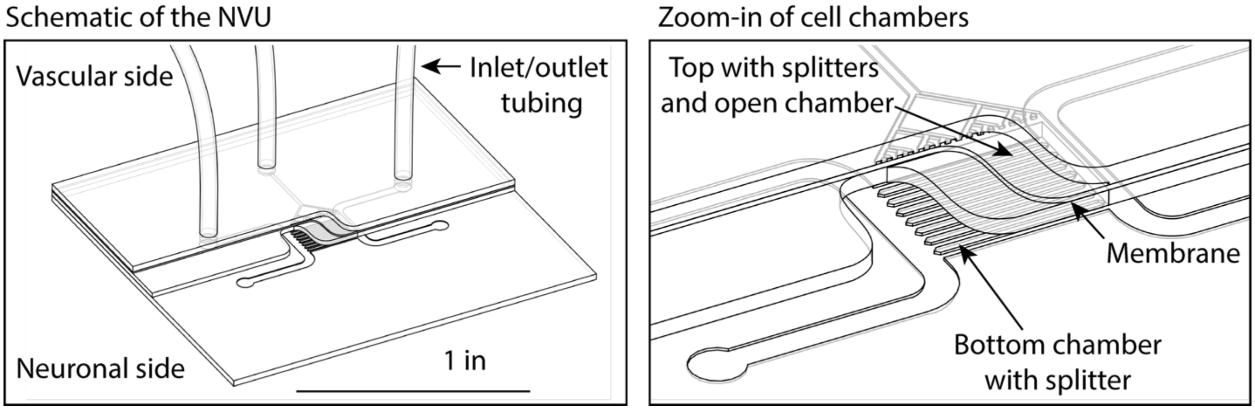

Supplemental Figure 4. Schematic of NVU design drawn to scale in CAD showing the full device (left) and a zoom in of the two chambers (right).

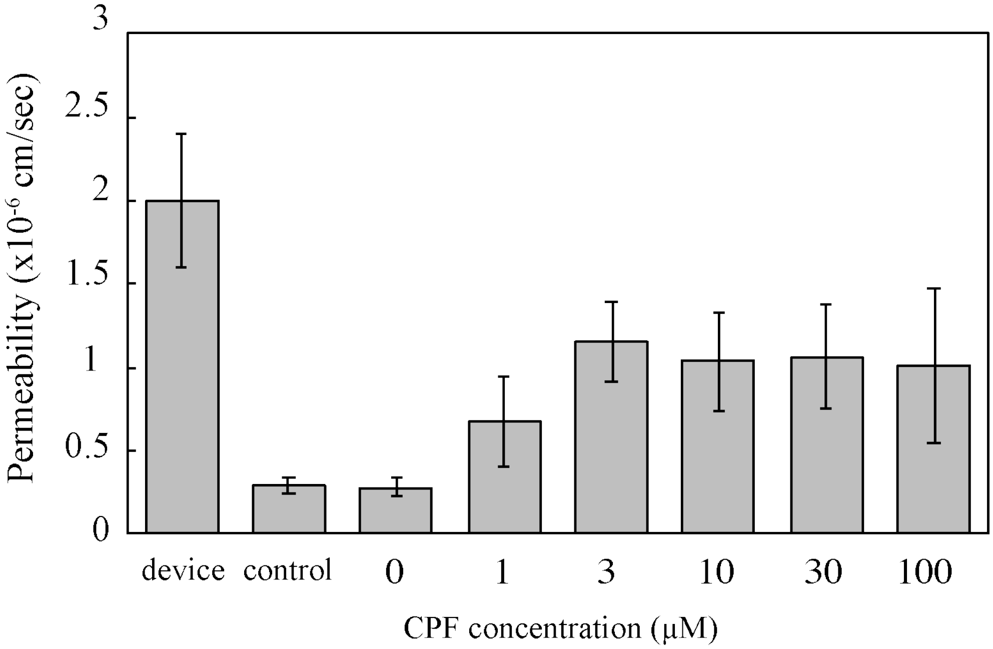

Supplementary Figure 5. Comparison of FITC permeability across the membrane of the NVU. Permeability of NVUs exposed to an escalating dose of CPF (0, 1, 3, 10, 30, 100 µM) are compared to a control NVU and an NVU device with no cells. FITC permeability was highest in the device with no cells (left bar). A control device was run in parallel with the test devices and the average permeability over time was similar to that of the test devices before CPF administration. Permeability of test devices increased after treatment with 1 µM CPF and stayed elevated over the course of the experiment. Data represented as mean and standard error. For test NVUs, n=5. For control NVU, n=5 (data points from different times). For empty device, n=3.

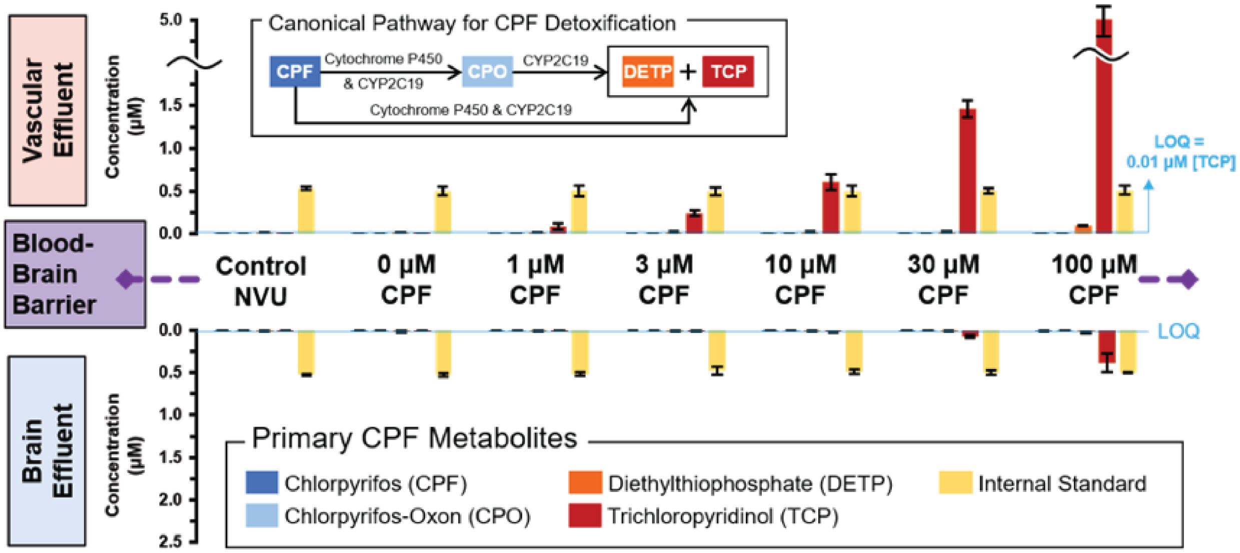

Supplementary Figure 6. Distribution of CPF and its three primary metabolites on and across the BBB with escalating doses of CPF. Using targeted MS, CPF and its three primary metabolites are quantified in both the vascular (**upper plots**) and neuronal (**lower plots**) eluate media samples. These samples were obtained with escalating doses of CPF (0, 1, 3, 10, 30 and 100 µM) within each NVU. Media samples were collected from both chambers of each NVU prior to each exposure event and stored in the freezer. For MS analysis, samples were exposed to cold ethanol for protein precipitation and centrifuged (15 minutes, 15,000 rpm) to remove cellular debris. Calibration curves were generated using serial dilutions of each primary metabolite, CPF, CPO, DETP, and TCP. An internal standard, DtBP, was also added to each sample to calibrate instrument response during each sample injection. Each dosing schedule contains eight replicate measurements (4 NVU devices, 2 technical replicates per sample) except the control NVU which contains four replicate measurements (2 NVU devices, 2 technical replicates per sample).

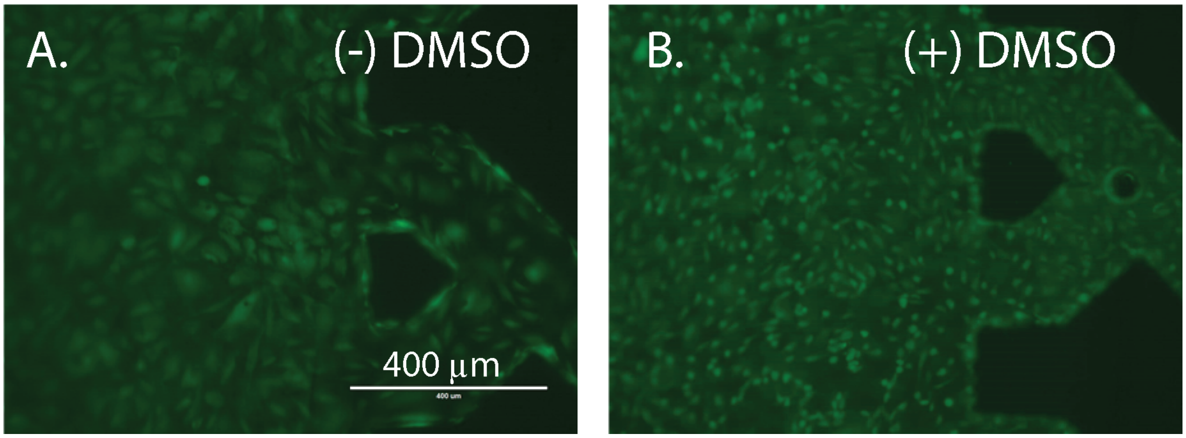

Supplementary Figure 7. Representative microscopy images of endothelial cells within an NVU following 24 hours of continuous exposure with either (**A**) media or (**B**) vehicle control (DMSO) slowing elongated cells in both instances. For these experiments, the NVUs were perfused with

neurobasal media on the neuronal side and EBM2 media on the vascular side either with or without DMSO. All cultures were maintained at 37ºC and 5% CO_2. Images courtesy of Jacquelyn A. Brown, Ph.D.

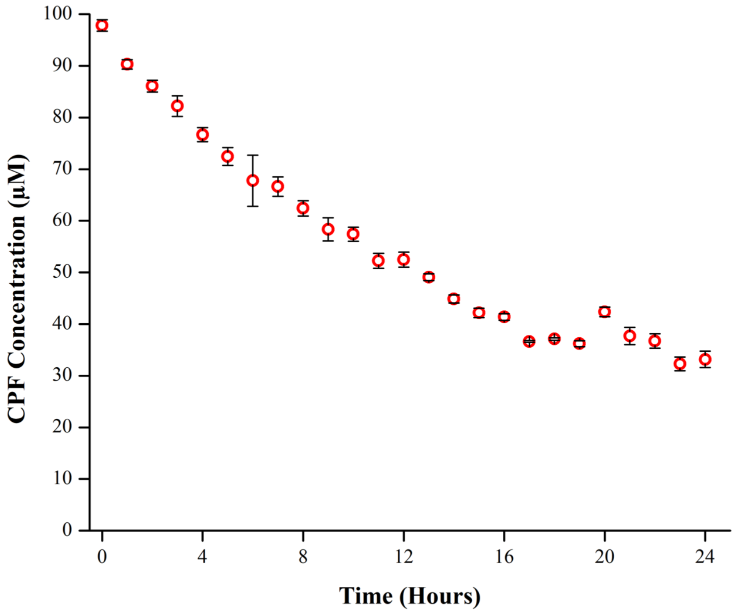

Supplemental Figure 8. PDMS absorption of CPF over 24 hours. PDMS disks (3 mm x 6 mm) were submerged in 100 µM CPF with 10 mM SDS (2 mL) for 24 hours. CPF remaining in solution was monitored every hour by measuring the absorbance at 290 nm. To determine the concentration of CPF remaining, a calibration curve was made with known concentrations of CPF (25-100 µM) and the unknown concentration was determined using the equation of the best fit line. All experiments were done in ambient conditions. Data shown as average and standard error, n = 3.
